# CircRNF10 triggers a positive feedback loop to facilitate progression of glioblastoma via redeploying the ferroptosis defense in GSCs

**DOI:** 10.1186/s13046-023-02816-9

**Published:** 2023-09-19

**Authors:** Chengbin Wang, Minjie Zhang, Yingliang Liu, Daming Cui, Liang Gao, Yang Jiang

**Affiliations:** 1 Department of Neurosurgery, Shanghai Tenth People’s Hospital, Tongji University School of Medicine, Shanghai, 200072 China; 2 Department of Neurology, Shanghai Tenth People’s Hospital, Tongji University School of Medicine, Shanghai, 200072 China

**Keywords:** GSCs, Ferroptosis, CircRNF10, ZBTB48, HSPB1

## Abstract

**Background:**

Glioma exhibit heterogeneous susceptibility for targeted ferroptosis. How circRNAs alterations in glioma promote iron metabolism and ferroptosis defense remains unclarified.

**Methods:**

The highly enriched circRNAs in glioblastoma (GBM) were obtained through analysis of sequencing datasets. Quantitative real-time PCR (qRT–PCR) was used to determine the expression of circRNF10 in glioma and normal brain tissue. Both gain-of-function and loss-of-function studies were used to assess the effects of circRNF10 on ferroptosis using in vitro and in vivo assays. The hypothesis that ZBTB48 promotes ferroptosis defense was established using bioinformatics analysis and functional assays. RNA pull-down and RNA immunoprecipitation (RIP) assays were performed to examine the interaction between circRNF10 and target proteins including ZBTB48, MKRN3 and IGF2BP3. The posttranslational modification mechanism of ZBTB48 was verified using coimmunoprecipitation (co-IP) and ubiquitination assays. The transcription activation of HSPB1 and IGF2BP3 by ZBTB48 was confirmed through luciferase reporter gene and chromatin immunoprecipitation (ChIP) assays. The stabilizing effect of IGF2BP3 on circRNF10 was explored by actinomycin D assay. Finally, a series of in vivo experiments were performed to explore the influences of circRNF10 on the glioma progression.

**Results:**

A novel circular RNA, hsa_circ_0028912 (named circRNF10), which is significantly upregulated in glioblastoma tissues and correlated with patients’ poor prognosis. Through integrated analysis of the circRNA-proteins interaction datasets and sequencing results, we reveal ZBTB48 as a transcriptional factor binding with circRNF10, notably promoting upregulation of HSPB1 and IGF2BP3 expression to remodel iron metabolism and facilitates the launch of a circRNF10/ZBTB48/IGF2BP3 positive feedback loop in GSCs. Additionally, circRNF10 can competitively bind to MKRN3 and block E3 ubiquitin ligase activity to enhance ZBTB48 expression. Consequently, circRNF10-overexpressed glioma stem cells (GSCs) display lower Fe^2+^ accumulation, selectively priming tumors for ferroptosis evading.

**Conclusion:**

Our research presents abnormal circRNAs expression causing a molecular and metabolic change of glioma, which we leverage to discover a therapeutically exploitable vulnerability to target ferroptosis.

**Supplementary Information:**

The online version contains supplementary material available at 10.1186/s13046-023-02816-9.

## Background

Glioblastoma multiform (GBM) is the most common primary malignancy of the central nervous system. Despite aggressive treatment measures, including maximal safe surgical resection and simultaneous radiotherapy and chemotherapy, the median survival of patients is only 14.6 months [1]. The unique biological properties of GBM, such as intratumoral and intertumoral heterogeneity [2], quiescent cancer stem cells [3], and intricate tumor immune microenvironment [4], pose significant challenges for the exploration of new treatment strategies [5]. Glioma stem cells (GSCs) are a class of GBM cells with self-renewal, tumor initiation, and clonal proliferation capacities. GSCs are strongly linked to the heterogeneity of GBM, and play a crucial role in clinical phenomena such as tumor recurrence, metastasis, and chemoradiotherapy tolerance [6].

Circular RNA (circRNA) is a class of structurally particular non-coding RNA that forms a closed-loop structure through covalent back-splicing without the 5' cap and 3' polyadenylation tail typical of linear RNAs. Compared to traditional linear RNAs, circRNAs exhibit strong resistance to degradation by ribonucleases (RNases) and therefore are expressed stably in cytoplasm [7]. Additionally, circRNAs are widely expressed in all eukaryotes and reveal high specificity in expression in different tissues and developmental stages. These features make circRNAs potentially powerful tools for diagnosis and prognosis [8]. Multiple studies related to malignant tumors have reported abnormal expression of circRNAs playing a significantly regulatory role in the processes of tumor proliferation, metastasis, drug resistance and recurrence [9]. For example, circPVT1 is highly expressed in breast cancer and promotes breast cancer development by targeting both ESR1 mRNA and MAVS protein [10]. CircEIF4G3 exerts a tumor-suppressive role in gastric cancer cells by regulating the stability of δ-catenin and the activity of miR-4449/SIK1 axis [11]. In the study of glioma and GBM, circASAP1 competitively binds to miR-502-5p contributing to dysregulation of NRAS, which activates the MEK1/ERK1-2 signaling pathway and promotes resistance to temozolomide [12]. Circ-E-Cad encoding peptide C-E-Cad activates EGFR/ EGFRvIII and promotes self-renewal of GSCs via both autocrine and paracrine signaling [13].

Ferroptosis is an emergingly discovered regulated cell death process in recent years and is significantly different from other programmed cell death pattern such as necrosis, apoptosis, autophagy, and pyroptosis in terms of cell morphology, molecular mechanisms, and biochemistry [14]. Ferroptosis occurs depending on the accumulation of ferrous ions, which mediates the aggregation of membranous peroxidized lipids, causing membranous instability or even rupture and ultimately leading to cell death. The metabolism of cancer cells is largely remodeled to satisfy increased bioenergetic and biosynthetic requirement and to support vigorous proliferation [15]. This metabolic reprogramming often has characteristic molecular features such as enrichment of polyunsaturated fatty acids and iron overload, leading to an imbalanced ferroptosis defense system, creating targetable vulnerabilities to ferroptosis and providing novel strategy for cancer treatment. The extent to which ferroptosis impacts cancer biology is unclear, although several studies have indicated that ferroptosis plays a main character in killing tumor cells and inhibiting tumor progression [16]. For instance, erastin, as a ferroptosis inducer, can enhance the sensitivity of GBM to temozolomide by restraining the function of cysteine/glutathione antiporter system Xc^−^ (xCT) and cystathionine-γ-lyase [17]. CircCDK14 expedites ferroptosis resistance and promotes tumor progression by regulating the expression of PDGFRA in GBM [18]. Herein, it is crucial to reveal the specific molecular mechanism of ferroptosis regulating by circRNAs and describe the crosstalk between ferroptosis and circRNAs in glioma or GBM.

ZBTB48 is a transcription factor containing a classical zinc finger structure. It has been renamed as telomeric zinc finger-associated protein (TZAP) due to its function in regulating telomere length [19]. Several investigations have demonstrated that TZAP binds directly to TTAGGG telomere repeats and replaces telomere repeat binding factors 1 and 2 (TRF1/2) as the telomere capping protein [20]. However, the transcriptional regulation function of ZBTB48 has not been reported, and the mechanism of its action in glioblastoma remains to be further studied and explored.

In current study, we primarily discovered a novel circular RNA, hsa_circ_0028912 (named circRNF10), which is significantly upregulated in glioblastoma tissues and positively correlated with poor prognosis in glioma patients. Our study aimed to elucidate the possible mechanisms by which circRNF10 regulates ferroptosis of GSCs, while revealing the concrete process circRNF10 affects ubiquitination-mediated stability of target protein. In addition, we also described the consequence of the circRNF10-ZBTB48-IGF2BP3 positive feedback loop in tumorigenesis and progression of GBM.

## Methods

### Glioma tissue specimen collection and ethical approval

This study involved a total of 70 glioma tissue specimens, all of which were obtained from glioma patients undergoing surgery at the Department of Neurosurgery, Shanghai Tenth People's Hospital between January 2015 and December 2019. All pathological specimens underwent histopathological examination and were graded according to the classification guidelines of the World Health Organization. Among them, there were 18 cases of grade 2, 24 cases of grade 3, and 18 cases of grade 4 gliomas. In addition, we collected 10 normal brain tissue specimens from GBM patients as normal control group. All enrolled cases signed informed consent, and this study was approved by the Ethics Committee of Shanghai Tenth People's Hospital.

### Cell culture and reagents

Four patient-derived GSCs were obtained and validated from four GBM specimens (SYNS03, SYNS05, SYNS06, and SYNS08) using the isolation, culture, and identification protocols described previously. The clinical information of the four GBM specimens is presented in detail in the Table S1. In brief, fresh tumor tissue obtained during surgery was mechanically dissociated and collagenase-digested to dissociate into single cells. The tumor cells were then suspended in StemFlex™ medium (Gibco, Gaithersburg, MD, USA) at 37℃ with 5% CO_2_ atmosphere and cultured for two weeks until tumor spheres formed. The stemness characteristics of GSCs were characterized by detecting the expression of stem cell markers, such as CD133, NESTIN, and SOX2, and verifying multi-lineage differentiation ability. All GSCs cell lines underwent short tandem repeat (STR) DNA analysis and mycoplasma detection. All GSCs used in the experimental research were capable of at least 20 passages. During the experimental assay, GSCs were processed with several reagents, including necrostatin-1 (an inhibitor of necroptosis, 20 μM), Z-VAD-FMK (a pan-caspase inhibitor, 20 μM), 3-Methyladenine (3-MA, an inhibitor of autophagy, 60 μM), and ferrostatin-1 (Fer-1, a ferroptosis inhibitor, 10 μM). Above all reagents were purchased from Sigma-Aldrich® (Merck, Germany).

### RNA fluorescence in situ hybridization (FISH)

FISH was performed using the FISH Tag™ RNA Multicolor Kit (Invitrogen, USA) according to the manufacturer's instructions. Oligonucleotide probes complementary to the circRNF10 junction sequence were synthesized by Gene-Chem (Shanghai, China). GSCs were seeded onto glass coverslips pre-treated with poly-D-lysine (Sigma-Aldrich). Images were acquired with a precision imaging system constituted by an ECLIPSE Ts2 fluorescence microscope, Digital Sight 10 camera and NIS-Elements software (Nikon, Japan).

### Construction and transfection of lentiviral vectors

The lentiviral vectors for the permanent overexpression of CircRNF10, ZBTB48, MKRN3, HSPB1, and IGF2BP3 and their corresponding negative controls were synthesized by Gene-Chem (Shanghai, China). Lentiviral vectors for RNAi-mediated knockdown of the above genes were also provided by Gene-Chem. The RNAi sequences used in this study are listed in the Table S2. The transfection efficiency of lentiviruses in GSCs was verified by qPCR and western blotting assays.

### Quantitative real‑time PCR (RT‑qPCR) assay

The total RNA was extracted using TRIzol (Invitrogen, USA) following the manufacturer's instructions. The cDNA library was constructed using the Prime-Script RT Master Mix Kit (TaKaRa, Kyoto, Japan). RT‑qPCR was performed using the SYBR Green Master Mix Kit (TaKaRa) on the Mx-3000P Quantitative PCR System (Applied Biosystems, USA). The obtained data were calculated and processed using the 2^−ΔΔCt^ method, with GAPDH expression as an endogenous control, to manifest the relative expression levels of genes. The primers used in this study were purchased from Sangon Biotech (Shanghai, China) and the sequences are listed in the Table S3.

### RNase R assay

RNase R has the activity of degrading linear RNAs, but is ineffective against circRNAs, and is used to ascertain the circular structure of circRNAs. 10 μg RNA extracted from GSCs were incubated with 40U RNase R (Epicentre Technologies, Madison, WI, USA) at 37 °C for 30 min. After the reaction completed, the expression of linear RNAs and circular RNAs were detected via RT-qPCR.

### Cytoplasmic and nuclear RNA separation assay

The isolation and purification of both cytoplasmic and nuclear RNA of GSCs was conducted using the Cytoplasmic & Nuclear RNA Purification Kit (NGB-21000, Norgen Biotek). Briefly, according to the manufacturer's instructions, GSCs were lysed with lysis buffer J, and different components were separated by centrifugation. The pellet fraction contained the cell nuclei, while the supernatant fraction contained the cytoplasm. Following the addition of Buffer SK to these fractions, pure RNA can be obtained through sequential washing and purification steps.

### Western blotting assay

GSCs from diverse groups were lysed by RIPA buffer containing 1% protease and phosphatase inhibitors (Beyotime Biotechnology, Beijing, China). The proteins were quantified and denatured, separated by SDS-PAGE gel electrophoresis and transferred onto PVDF membranes. The membranes were then incubated with the primary antibody against the target protein at 4 °C overnight. Next, the primary antibody was washed away and the secondary antibody was added and incubated at room temperature for 1 h. Protein bands were visualized using the chemiluminescence ECL kit (YEASEN, Shanghai, China) and a chemiluminescence imaging system (Tanon, Shanghai, China). The gray values of the protein bands were quantified by ImageJ software (National Institutes of Health, Bethesda, MD, USA). The antibodies used in this study were listed in the Table S4.

### Protein stability assessment

GSCs from various groups were preconditioned with 50 μM MG132 (Sigma-Aldrich), a proteasome inhibitor, for 6 h during the cultivation process. After protein extraction, western blotting analysis was performed to detect the target proteins. In addition, GSCs were treated by 50 μg/ml cycloheximide (Sigma-Aldrich), an inhibitor for neo-proteins synthesis, and lysed to harvest proteins at 0 h, 2 h, 4 h, 8 h, and 12 h for protein half-life assay through western blotting detection.

### RNA pull-down assay and mass spectrometry

The RNA pull-down assay was performed using the Pierce™ Magnetic RNA–protein pull-down kit (Thermo Fisher Scientific, USA) according to the manufacturer's instructions. Briefly, the lysate of GSCs was incubated with biotinylated circRNF10, anti-sense or mutant probes. Then, streptavidin-conjugated magnetic beads were added into the system. After elution and purification, the sorted proteins were separated by SDS-PAGE gel electrophoresis and visualized using a silver staining kit (YEASEN) for subsequent mass spectrometry analysis, and the expression levels of interactional proteins were confirmed by western blotting. The protein band stained with silver was excised for digestion, and then analyzed using a QExactive mass spectrometer (Thermo Fisher Scientific).

### Co‑immunoprecipitation (Co-IP)

The Co-IP assays were analyzed using the Pierce Classic Magnetic IP/Co-IP Kit (Thermo Fisher Scientific). GSCs were lysed with IP lysis buffer containing protease and phosphatase inhibitors (leupeptin, aprotinin and phenylmethylsulfonyl fluoride). According to the manufacturer’s protocol of co-IP kit, the GSCs lysates were incubated with antibodies coupled magnetic beads at 4 °C overnight. Immunoprecipitates and whole-cell lysates were collected for western blotting to analyses the interactive ability of proteins.

### In vivo ubiquitination assay

The ubiquitination assay was performed as previously described [21]. GSCs of different groups were transfected with Flag-ZBTB48, HA-Ub-WT, HA-Ub-K6R, K11R, K27R, K29R, K33R, K48R, or K63R plasmids (Gene-Chem) using Lipofectamine 3000 (Invitrogen) before treatment with MG132 for 6 h. Lysates of GSCs were prepared by IP buffer and collected for ubiquitination assay. Antibody against the Flag tag (Abcam Technology, Cambridge, UK) was used to proteins immunoprecipitation, and then the ubiquitination was detected by immunoblotting with anti-HA or anti-Ubiquitin antibodies (Abcam).

### RNA immunoprecipitation (RIP) assay

EZ-Magna RIP kit (Millipore, Darmstadt, Germany) was leveraged for RIP assay according to manufacturer’s protocols. Briefly, GSCs were lysed with PIP buffer and incubated with anti-ZBTB48, anti-MKRN3, and anti-IGF2BP3 antibodies conjugated to magnetic beads. The protein-RNAs complexes were immunoprecipitated, and RNAs were collected after proteinase K treatment, washing and purification steps. The expression level of circRNF10 was measured by RT-qPCR. IgG antibody (Abcam) was used as a negative control in this experimental assay.

### Cell proliferation assay

Cell proliferation was evaluated using the MTS assay. GSCs were seeded into 96-well plates at a 10^3^ cells /well density, and 20 μl of MTS (Promega, Madison, WI, USA) was added to each well at 24, 48, 72, 96, and 120 h. After incubating for 3 h, absorbance at 490 nm was measured using a UV spectrophotometer (Thermo Fisher Scientific). In addition, the EdU assay was also used to evaluate the proliferation capacity of GSCs. The EdU assay was performed by using the EdU assay kit (Beyotime) according to the experimental protocols. Briefly, GSCs were seeded at a 10^4^/well density in 24-well plates and cultured for 24 h. Then, 10 μM of EdU reagent was added to each well and incubated for 2 h. After steps of fixing, EdU and nuclear staining of the GSCs, image acquisition of the experimental results was completed using an ECLIPSE Ts2 fluorescence microscope (Nikon, Japan). Finally, the percentage of EdU-positive cells was calculated.

### Neurosphere formation assay (NSFA)

GSCs were cultured in suspension in 6-well plates at a density of 10^3^ cells/well. StemFlex™ medium (Gibco) 1.5 ml was added to each well and continuously cultured for 7 days. The images of the neurospheres were obtained using an optical microscope (Olympus), and relative diameters were recorded for subsequent analysis calculations.

### Extreme limiting dilution assay (ELDA)

Well-grown GSCs were collected and seeded in 96-well plates at densities of 1, 10, 20, 30, 40, and 50 cells per well. Each density was repeated ten times and continuously cultured for 7 days. The number of wells with neurospheres formation (NSF) were observed and recorded using an inverted optical microscope (Olympus). The neurosphere synthesis capacity of GSCs were analyzed via using Extreme Limiting Dilution Analysis (ELDA, http://bioinf.wehi.edu.au/software/elda).

### Lipid peroxidation assessment

The level of lipid peroxidation in GSCs was evaluated using the BODIPY 581/591 C11 kit (Thermo Fisher Scientific). Briefly, according to the manufacturer's instructions, GSCs were stained with 2 μM C11-BODIPY (581/591) probe. The oxidized BODIPY (O-BODIPY) and the reduced BODIPY (R-BODIPY) were visualized at excitation/emission wavelengths of 488/510 (traditional FITC filter set) and 581/591 nm (Texas Red filter set), respectively, utilizing a confocal microscope (LSM 880; Carl Zeiss AG, Jena, Germany). The relative fluorescence intensity of the C11-BODIPY probe was analyzed using Image J software (NIH, Bethesda, MD, USA).

### Measurement of intracellular Fe^2+^

The concentration of intracellular labile ferrous (Fe^2+^) ions in GSCs was detected using the BioTracker FerroOrange kit (Sigma-Aldrich). FerroOrange is a live cell imaging probe that specifically detects Fe^2+^ ions. Briefly, according to the manufacturer's instructions, GSCs were incubated with 1 μM FerroOrange probe at working solution and observed under a confocal microscope (Carl Zeiss) at excitation/emission wavelengths of 581/591 nm (Texas Red filter set). The relative fluorescence intensity of the FerroOrange probe was analyzed using Image J software (NIH, Bethesda, MD, USA).

### Glutathione (GSH) and malondialdehyde (MDA) assay

The Reduced Glutathione (GSH) Assay Kit (Colorimetric, Sigma-Aldrich) was used to detect the GSH content in GSCs. According to the manufacturer's protocols, GSCs were harvested and gradually reacted with the reagents. Finally, the GSH content was reflected by measuring the absorbance of the reaction products at 450 nm using a UV spectrophotometer (Thermo Fisher Scientific). Similarly, the malondialdehyde (MDA) content in GSCs was detected using the Lipid Peroxidation (MDA) Assay Kit (Colorimetric/Fluorometric, Abcam). In the lipid peroxidation assay protocol, the MDA in the GSCs reacts with thiobarbituric acid (TBA) to generate the MDA-TBA adduct quantified colorimetrically at 532 nm.

### Dual luciferase reporter gene assay

The purpose of the dual-luciferase reporter gene assay was to analyze the transcriptional regulation of transcription factors on target genes. The complete nucleotide sequences of the HSPB1 and IGF2BP3 promoters as wide- type (HSPB1-WT, IGF2BP3-WT), and ZBTB48 binding site mutant clones of the promoter regions (HSPB1-MT, IGF2BP3-MT) were cloned into the pGL3 vectors. GSCs were seeded in a 24-well plate and co-transfected with pRL TK (renilla luciferase reporter vector) and pGL3 vector plasmids (or HSPB1-WT, HSPB1-MT, IGF2BP3-WT, IGF2BP3-MT plasmids). After 24 h, the activity of luciferase and renilla luciferase was detected and analyzed using a dual-luciferase reporter gene assay kit (Beyotime) according to the operation manual.

### Chromatin immunoprecipitation (ChIP) assays

ChIP assays were performed using the ChIP Assay Kit (Beyotime) according to the manufacturer’s instructions. Briefly, an anti-ZBTB48 antibody, Histone H3 antibody (Cell signaling technology) as a positive control and IgG antibody (Abcam) as a negative control were used to immunoprecipitate the chromatin complexes. Then DNA was extracted and purified from the complexes and analyzed by qPCR. The primers for ChIP qPCR are listed in Table S5.

### RNA stability assay

GSCs with knockdown or overexpression of IGF2BP3 were treated with actinomycin D (Sigma-Aldrich) at a final concentration of 5 μg/mL for 2, 4, 6, 8, and 10 h. Total RNA of GSCs was extracted and the content of circRNF10 was detected by qPCR to calculate the RNA turnover rate and half-life (t_1/2_).

### Cell death kinetics assay

The cell death kinetics assay of GSCs was conducted using the Nikon laser confocal microscope AX/AX R imaging system. GSCs were seeded in 24-well culture plates at a well-established growth state of 10^5^ cells/well. The different research groups of GSCs were treated and incubated with propidium iodide (PI) staining solution (BD Bioscience, USA) following the provided instructions. Real-time fluorescence and differential interference contrast imaging of cells in the culture plates were performed every 2 h from 0 to 48 h and the cell death kinetic curve was calculated based on counting and analyzing the PI-positive dead cells.

### Cell cycle assay

GSCs were harvested and fixed in 70% ethanol for 24 h. The GSCs were then centrifuged, resuspended and stained with PI. The cell-cycle assay was conducted leveraging the BD Accuri™ C6 Plus flow cytometer.

### Transmission electron microscopy

GSCs were fixed and collected using electron microscopy fixative. The samples were then transported to the School of Life Sciences and Technology of Tongji University for transmission electron microscopy analysis.

### Xenograft mice experiment

The animal experimental protocol was approved by the Ethics Committee of Laboratory Animals of the Shanghai Tenth People's Hospital. 6-week-old female immunodeficient NOD/SCID mice were purchased from the Model Animal Research Center of Nanjing University (Nanjing, China). All mice were raised under specific pathogen-free conditions at the Laboratory Animal Center of the Shanghai Tenth People's Hospital. Each group included 5 mice for the establishment of orthotopic glioblastoma transplantation models. After anesthesia, GSCs were injected into the mice brain at a density of 5 × 10^4^ cells in 5μL solution using a stereotactic apparatus by puncturing at the 2 mm lateral and anterior to intersection of the coronal and sagittal sutures on the skull. The survival time of each group of mice were recorded and the brains were collected after transcadiac perfusion with 4% paraformaldehyde for H&E and immunohistochemical (IHC) staining assay. The formula used for calculating tumor volume was as follows: V = (D × d ^2^) / 2, with D being the maximum diameter and d being the minimum diameter.

### Immunohistochemistry (IHC) and immunofluorescence (IF) assay

IHC staining of tumor sections from GSCs xenografted mice brain were performed using an immunohistochemical labeling kit (Immunoway Biotechnology, USA) according to the manufacturer’s protocol. The stained images of tumor tissue sections are obtained through upright microscopy (Leica, DM4000B), and the staining results are quantified according to the German IHC scoring system. For the IF staining assay of GSCs, briefly, GSCs were seeded onto glass coverslips pre-treated with poly-D-lysine (Sigma-Aldrich). Growth status of well-cultured GSCs was assessed by fixing, permeabilizing, and antigen blocking. The cells were then incubated with the primary antibody at 4 °C overnight. Subsequently, a secondary antibody labeled with a fluorescent probe was added and incubated at room temperature for 1 h. Cell nuclei were stained with DAPI dye (Abcam). Finally, experimental results were visualized using an ECLIPSE Ts2 fluorescence microscope (Nikon, Japan).

### Bioinformatics analysis

The biological information of CircRNF10 was obtained from circBase (http://www.circbase.org), Cancer-Specific CircRNA Database (CSCD, https://gb.whu.edu.cn/CSCD/), and circInteractome (http://circinteractome.nia.nih.gov). The iron death-related gene set was obtained from Gene Set Enrichment Analysis (GSEA, http://www.broadinstitute.org/gsea/index.jsp). The transcriptome level data of ZBTB48 and HSPB11 in the TCGA glioma cohort were obtained from the GDC Data Portal (https://portal.gdc.cancer.gov/). Similarly, RNA-seq data of the Chinese Glioma Genome Atlas (CGGA) were downloaded from the CGGA database (http://www.cgga.org.cn/).

### Statistical analysis

All statistical analyses in this study were performed by using R version 4.2.1 and GraphPad Prism version 9.0 software. The Shapiro–Wilk normality test and Bartlett test were used to verify the normal distribution of acquired data and homogeneity of variances, respectively. T-tests were employed for comparisons of two independent samples, while one-way ANOVA was used for comparisons among multiple samples. For non-parametric data, the Wilcoxon test were performed, Pearson correlation algorithm was employed to assess the correlation between two or more groups. Finally, survival analysis between different groups were evaluated and visualized by performing the Kaplan–Meier curve and log-rank test. *P*-values < 0.05 were accepted as statistically significant (*p < 0.05; **p < 0.01; ***p < 0.001).

## Result

### Increased circRNF10 expression in glioma is correlated with poor prognosis

**Fig. 1:**
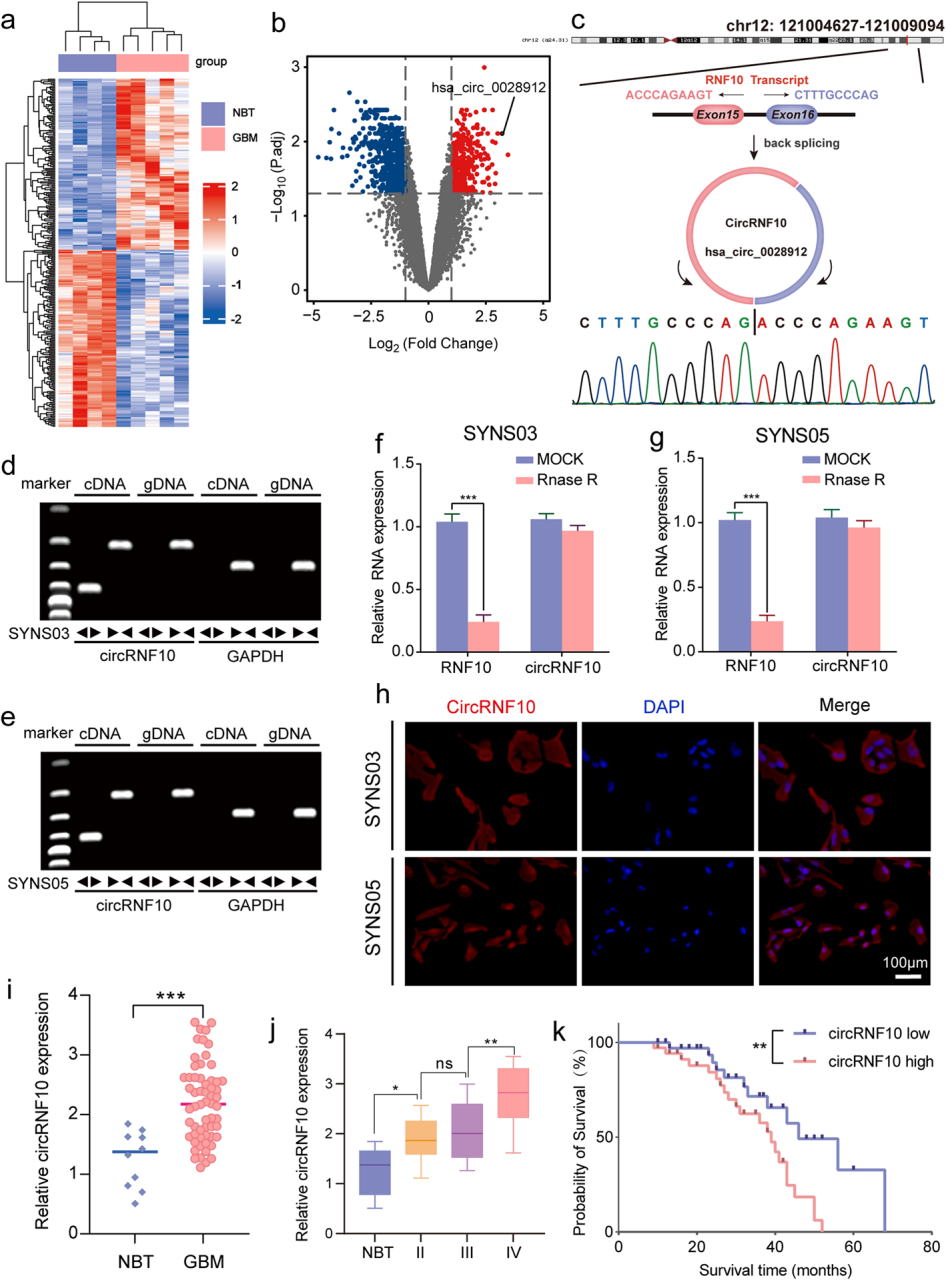
circRNF10 is upregulated in glioma and correlated with poor prognosis. **a** Heatmap displaying the z-scores value of circRNAs differentially expressed between normal brain tissue (NBT) and GBM in GSE165926. **b** Volcano plot depicting the log_2_(fold change) of circRNAs in the two types of tissue mentioned above. Grey dashed lines represent the cutoff value, that is, adjusted *P* value < 0.05, | log_2_[fold change] |> 1. Downregulated (blue) and upregulated (red) circRNAs are color-coded. **c** Schematic diagram of genomics information of circRNF10 (hsa_circ_0028912). Upper panel illustrated the maternal gene location and exon composition. And back splicing site identified by Sanger sequencing was shown in the bottom of the panel. **d**, **e** RT-qPCR analysis showing the expression of circRNF10 amplified from templates from the two GSC cell lines SYNS03 (**d**) and SYNS05 (**e**) using divergent and convergent primers. **f**, **g** The mRNAs level of circRNF10 and RNF10 in SYNS03 (**f**) and SYNS05 (**g**) after RNase R treatment. **h** In situ hybridization detection of circRNF10 mRNA expression in SYNS03 and SYNS05. Scale bar = 100 μm. **i** The circRNF10 expression level in glioma tissues (*n* = 70) and normal brain tissues (*n* = 10). **j** CircRNF10 expression in gliomas specimens with different malignant grades. **k** Kaplan–Meier survival curve for all glioma patients with high and low circRNF10 expression. Data are presented as means ± SD (three independent experiments). *p < 0.05; **p < 0.01; ***p < 0.001; ns, no significance

To investigate the aberrant expression of circRNAs in GBM, we searched the Gene Expression Omnibus (GEO) and identified the GSE165926 dataset containing circRNA sequencing results from both GBM and normal brain tissues. We further analyzed the differentially expressed circRNAs using the limma R package and seized 330 significantly upregulated circRNAs and 663 downregulated circRNAs (|Log2FC|> 1, *P*. adj < 0.05) in GBM compared to normal brain tissues (Fig. 1a, b). Considering that overexpression of circRNAs often promotes the malignant progression of gliomas, we first carefully sifted the top 10 circRNAs with the most significant expression differences regarding 330 abnormally high expressed circRNAs. Subsequently, we performed qPCR detection of these 10 circRNAs in a cohort of 70 glioma patients. Among the top 10 circRNAs, hsa_circ_0028912 displayed the highest expression level (Fig. S1). The circRNF10 with a circBase ID of hsa_circ_0028912 is located at chr12:121,004,627–121009094, and formed by back-splicing of the 15th and 16th exons of the RNF10 gene (Fig. 1c). The back-splice junction site of circRNF10 was amplified with divergent primers and confirmed through Sanger sequencing. qPCR assays were performed in cDNA reversely transcripted from SYNS03 and SYNS05, as well as genomic DNA (gDNA), using divergent and convergent primers to amplify circRNF10 separately. Analysis of qPCR products through agarose gel electrophoresis revealed that only divergent primers could amplify circRNF10 (Fig. 1d, e). Furthermore, RNase R treatment did not affect the expression levels of circRNF10 due to its closed loop structure, while linear RNF10 mRNA dropped notably (Fig. 1f, g). In SYNS03 and SYNS05, FISH analysis confirmed that circRNF10 mainly localized in the cytoplasm (Fig. 1h). The qPCR detection revealed that the expression of circRNF10 was markedly higher in GBM than in normal brain tissues, and its expression increased with the tumor grade (Fig. 1i, j). Kaplan–Meier survival analysis indicated patients with low circRNF10 expression had observably longer median survival time than patients with high circRNF10 expression (Fig. 1k). In conclusion, the above results suggest that circRNF10 is significantly overexpressed in GBM and is associated with poor prognosis of patients.

### Regulating circRNF10 impacts proliferation and stemness of GSCs in vitro

Given the expression and prognostic significance of circRNF10 in glioblastoma (GBM), we measured the expression levels of circRNF10 in GSCs from four GBM patients using qPCR. The results showed that the expression of circRNF10 in SYNS03 and SYNS08 was significantly higher than that in SYNS05 and SYNS06 (Fig. S2a). Therefore, SYNS03 and SYNS08 were selected for functional studies after circRNF10 knockdown, while SYNS05 and SYNS06 were designated for circRNF10 overexpression. The efficiency of circRNF10 overexpression was verified through qPCR detection (Fig. S2b). MTS assays indicated that circRNF10 overexpression in SYNS05 and SYNS06 obviously enhanced cell viability (Fig. S2c, d). Similarly, EdU analysis suggested that circRNF10 overexpression also elevated the proliferation ability of SYNS05 and SYNS06, as demonstrated by an increase in the percentage of EdU-positive cells (Fig. S2e, f). Besides, NSFA and ELDA analysis illustrated that the relative diameter and neurosphere-forming ability of SYNS05 and SYNS06 were increased after circRNF10 overexpression (Fig. S2g-j). We further determined the expression levels of stemness markers NANOG, OCT4, CD133, SOX2, and NESTIN in GSCs through western blotting, and the results reported the increased expression of stemness markers in SYNS05 and SYNS06 (Fig. S2k). Furthermore, cell cycle analysis indicated that circRNF10 overexpression facilitated the transition of SYNS05 and SYNS06 from G1 to S phase (Fig. S2l). Based on these related experimental results, circRNF10 has biological functions promoting the stemness maintenance and proliferation of GSCs.

Complementary to circRNF10 overexpression in GSCs, the effect of its downregulation was also assessed in SYNS03 and SYNS08 (Fig. S3a). Relevant molecular biology assays were conducted to further support the oncogenic effect of circRNF10. MTS, EdU, and NSFA all confirmed that circRNF10 silencing significantly reduced the cell viability, proliferation capacity, and neurosphere-forming ability of GSCs (Fig. S3b-i). Further western blotting analysis corroborated that the expression of stemness markers in GSCs was reduced observably after circRNF10 knockdown (Fig. S3j). Cell cycle analysis indicated that circRNF10 silencing inhibited the transition of SYNS03 and SYNS08 from G1 to S phase (Fig. S3k). The preliminary studies fully affirmed the significant role of circRNF10 silencing in suppressing the malignant phenotype of GSCs.

### CircRNF10 mediates ferroptosis- defending in GSCs

**Fig. 2:**
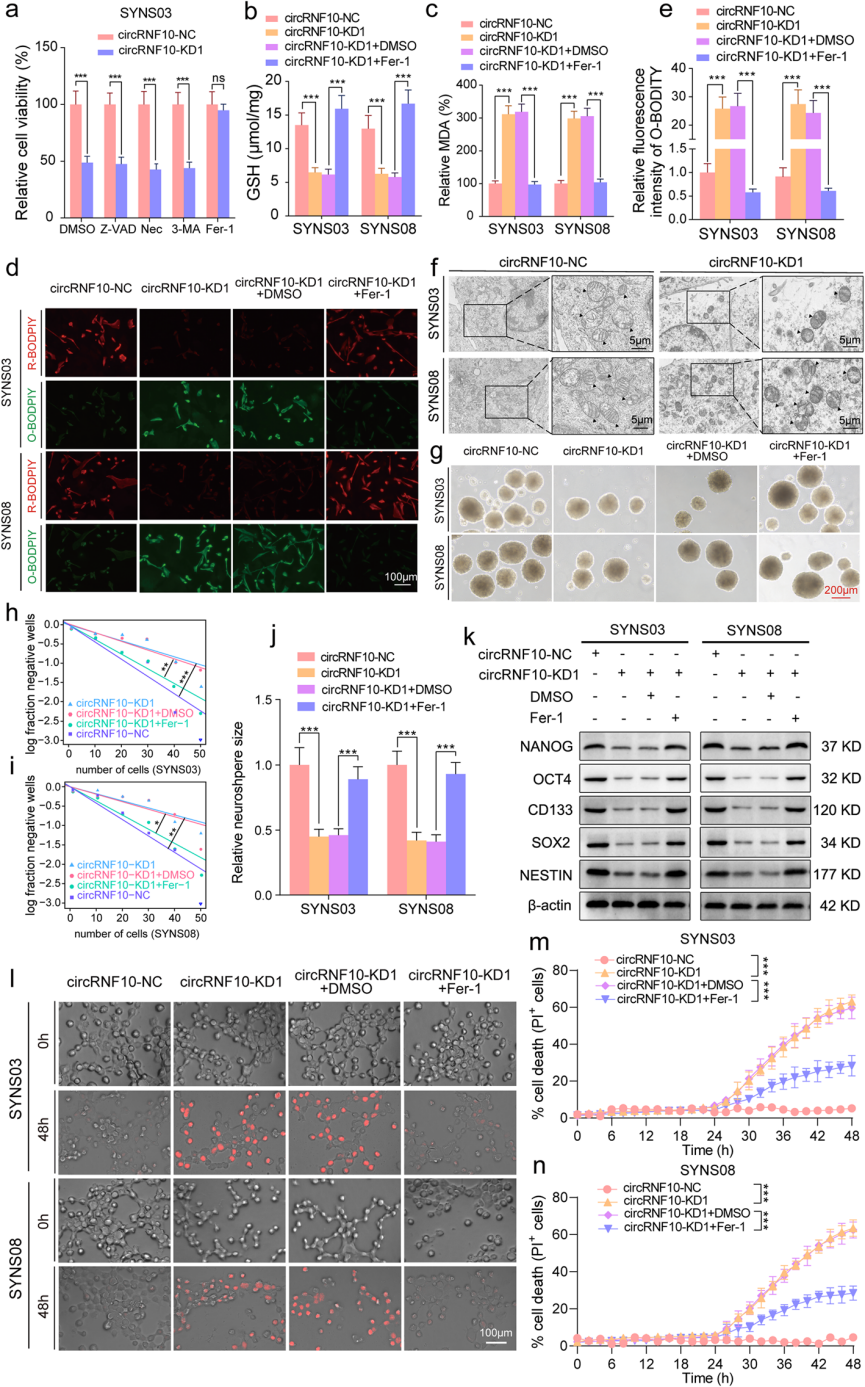
CircRNF10 promotes GSCs proliferation and stemness maintenance in a ferroptosis-defending manner in vitro. **a** Relative cell viability of SYNS03 treated with DMSO, Nec (20 μM), Z-VAD (20 μM), 3-MA (60 μM), and Fer-1(10 μM) for 24 h. **b**, **c** GSH(b) and MDA(c) contents measured in SYNS03 and SYNS08 with circRNF10 knockdown followed by Fer-1 treatment. **d**, **e** Lipid peroxidation levels (**d**) detected by BODIPY (581/591) C11 probe in SYNS03 and SYNS08 and the relative fluorescence intensity of O-BODIPY (**e**) quantified by Image J. Scale bar = 100 μm. **f** Morphology of mitochondria under transmission electron microscopy after circRNF10 knockdown in SYNS03 and SYNS08. Scale bar = 5 μm. **g** Representative images of NSFA with circRNF10 knockdown in SYNS03 and SYNS08 followed by Fer-1 treatment. Scale bar = 200 μm. **h**, **i** ELDA of neurospheres formation ability in SYNS03 (**h**) and SYNS08 (**i**) after circRNF10 silencing followed by Fer-1 treatment. **j** Relative sizes of neurospheres of circRNF10-silenced SYNS03 and SYNS08 followed by Fer-1 treatment. **k** Western blotting of the stemness markers expression with circRNF10 knockdown in SYNS03 and SYNS08 followed by Fer-1 treatment. **l** Representative images of cell death in SYNS03 and SYNS08 after 48 h of indicated treatments. Scale bar = 100 μm. **m**, **n** Real-time analysis of cell death in SYNS03 (m) and SYNS08 (n) with circRNF10 knockdown followed by Fer-1 treatment. Data are shown as the mean ± SD (three independent experiments). *p < 0.05; **p < 0.01; ***p < 0.001; ns, no significance

Compelling evidence from previous work has shown that regulated cell death (RCD) is characterized by explicit signaling pathways and plays prominent roles in tumor progression [22]. The novel RCD including necroptosis, pyroptosis, ferroptosis, and cuproptosis can occur with exogenous microenvironmental or intracellular perturbations. Malignant cells have also been reported to evade the RCD routes through evolving diversified mechanisms [23]. Based on the effect of circRNF10 on the phenotype of GSCs, we hypothesized that circRNF10 may regulate the RCD process of GSCs. Subsequently, various inhibitors of RCD were applied to treat circRNF10-silenced SYNS03. The results showed that only Fer-1 rescued the cell viability inhibition effect caused by circRNF10 silencing in SYNS03 (Fig. 2a), while suggesting that circRNF10 upregulation may confer on GSCs the ability to defend against ferroptosis and promote proliferation and stemness maintenance.

To further explore the role of circRNF10 silencing in the process of ferroptosis of GSCs, we first detected the level of reduced glutathione (GSH) in SYNS03 and SYNS08. The obtained data showed that the GSH content significantly decreased after circRNF10 knockdown, and this effect was tolerably abolished after Fer-1 treatment subsequently (Fig. 2b). In addition, malondialdehyde (MDA), as an oxidative metabolite, which is a popular indication of ferroptosis, was also assessed to inform the changes of oxidation products in the cytoplasm. The results displayed that the MDA content increased in SYNS03 and SYNS08 after circRNF10 silencing and was restored after Fer-1 treatment (Fig. 2c). Since ferroptosis is characterized by the accumulation of intracellular reactive oxygen species (ROS), in circRNF10-knockdown SYNS03 and SYNS08, BODIPY (581/591) C11 probe was added to detect the ROS level. The content of oxidized probe O-BODPIY increased under the experimental conditions. And similarly, Fer-1 intervention reduced the content of O-BODPIY and increase the content of reduced probe R-BODPIY (Fig. 2d, e). As there are morphological changes observed under optical microscope in cells undergoing ferroptosis, transmission electron microscopy was used to visualize the subcellular structure in SYNS03 and SYNS08. Consistent with the biochemical data, after circRNF10 knockdown, GSCs showed typical morphological features such as mitochondrial shrinkage, mitochondrial membrane folding, and increased membrane density (Fig. 2f). In the cellular ferroptosis defense mechanism, GPX4 and SLC7A11 indeed play important roles, especially in malignant tumors where aberrant expression or function of GPX4 and SLC7A11 often accompanies and affects the susceptibility of cancer cells to ferroptosis. We conducted further qPCR analysis on the collected clinical samples to explore the correlation between circRNF10 and GPX4/SLC7A11 expression. The results show a weak positive correlation between circRNF10 and GPX4 expression, and no correlation between circRNF10 and SLC7A11 expression (Fig. S4a, b). These consequences may suggest that circRNF10 may not defend against cellular ferroptosis through the regulation of the SLC7A11/GSH/GPX4 pathway. In summary, the above observations confirm that circRNF10 silencing can induce ferroptosis in GSCs.

### CircRNF0 promotes GSCs proliferation through suppressing ferroptosis in vitro

Based on results of ferroptosis morphology and related intracellular oxidative and reduction product indicators, circRNF10 has a regulatory role in ferroptosis of GSCs. However, it remains to be clarified whether circRNF10 promotes GSCs cell viability and accelerates proliferation by defending ferroptosis. To address this, MTS, EdU, NSFA, ELDA, and cell cycle assays were conducted on SYNS03 and SYNS08 with circRNF10 knockdown and Fer-1 treatment was simultaneously implemented. The measurements of MTS (Fig. S5a, b) and EdU (Fig. S5c, d) assays showed that the decrease of cell viability and proliferation capacity caused by circRNF10 knockdown in SYNS03 and SYNS08 can be rescued by adding Fer-1. This agreed with the NSFA and ELDA results that also indicated that Fer-1 administration counteracts the decreased neurosphere formation ability caused by circRNF10 silencing (Fig. 2g-j). Furthermore, Western blotting results for GSC stemness markers indicated that Fer-1 enhanced the impaired stemness maintenance ability caused by circRNF10 silencing in SYNS03 and SYNS08 (Fig. 2k). We further demonstrated the relationship between cancer stem cells and ferroptosis. In the TCGA dataset, we conducted Gene Set Variation Analysis (GSVA) to evaluate the ferroptosis pathway score (GSVA-FerroScore) for each glioma sample. Additionally, we obtained the stemness indices (mRNA-si) for each sample in the TCGA-GBMLGG dataset. We also performed a correlation analysis between GSVA-FerroScore and mRNA-si [24], and the results showed a significant negative correlation between the two scores (Fig. S5e). These results indicate that the stemness maintenance ability of cancer cells is complementary to their ferroptosis defense capacity. Hereafter, we observed that the cell cycle of SYNS03 and SYNS08 was arrested in the G1 phase caused by circRNF10 knockdown, which can be transited to the S phase by Fer-1 treatment (Fig. S5f). Similarly, the kinetics of cell death induced by knockdown of circRNF10 in GSCs could be dynamically alleviated by Fer-1 treatment (Fig. 2l-n). Together the above data demonstrate circRNF10 promotes GSCs proliferation and stemness maintenance by defending ferroptosis.

### Identification of ZBTB48 as the target of circRNF10 in GSCs

**Fig. 3:**
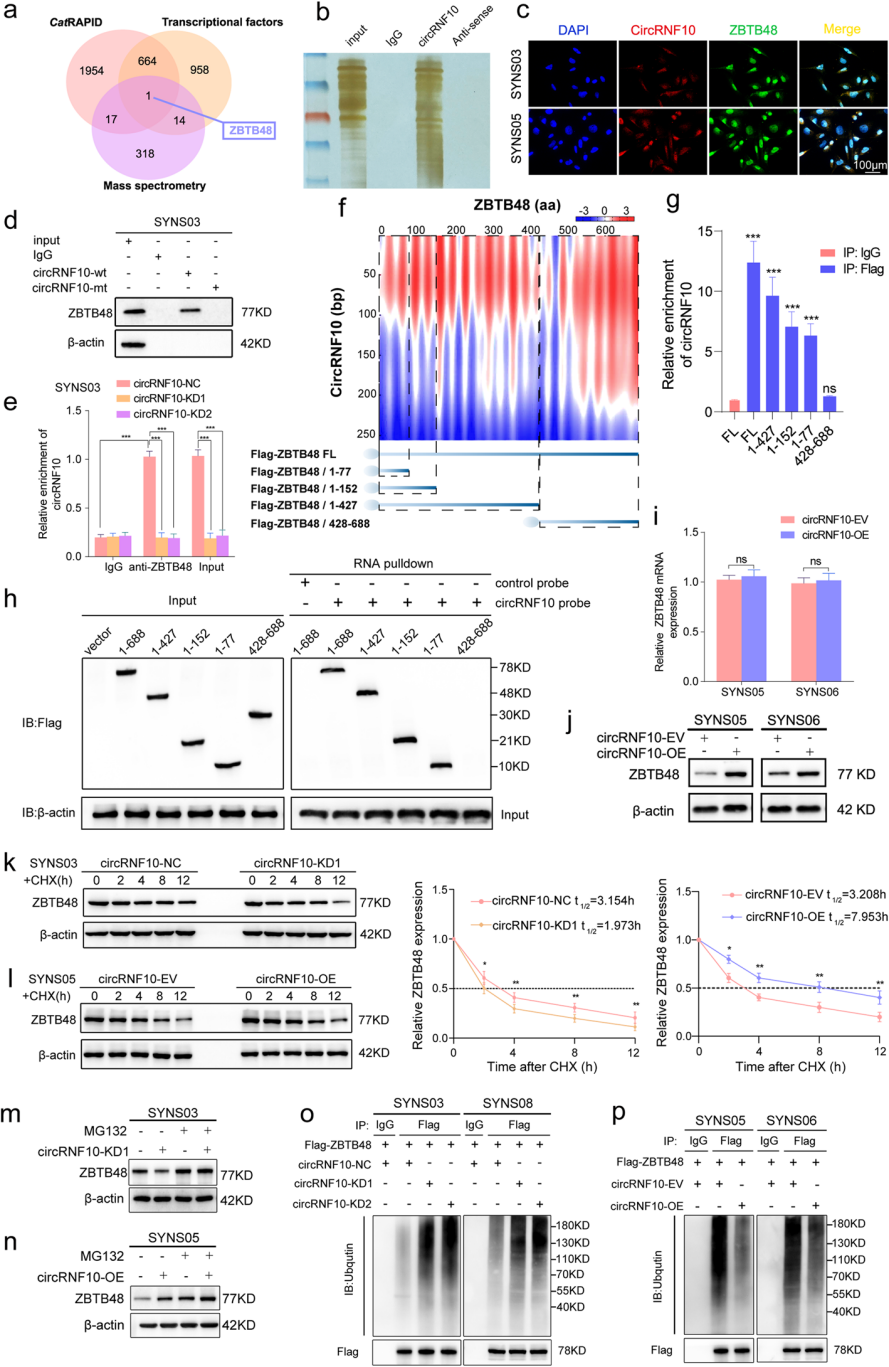
CircRNF10 interacts with ZBTB48 and prevents from degradation via inhibiting the ubiquitin- proteasomal pathway. **a** Venn chart of circRNF10 downstream target ZBTB48 screening. **b** Silver staining for circRNF10 interacting proteins in SYNS03 separated by SDS-PAGE. **c** Co-localization of circRNF10 (RED) and ZBTB48 (GREEN) respectively, in SYNS03 and SYNS05. Scale bar = 100 μm. **d** RNA pull-down assays in SYNS03 displayed the circRNF10 probe pulled down ZBTB48 proteins. **e** RIP assays showed anti-ZBTB48 treatment leaded to circRNF10 enrichment in circRNF10-silenced SYNS03. **f** RNA–protein interaction between circRNF10 and ZBTB48 predicted by the CatRAPID algorithm (top) and the diagrams of Flag-tagged ZBTB48 truncations (bottom). **g** RIP assays were performed in SYNS03 transfected with the truncated mutant vectors to validate the binding domain of ZBTB48. **h** Left, western blot showed the expression of Flag-tagged ZBTB48 truncations of SYNS03 transfected with the indicated vectors; Right, RNA pulldown assays revealed the enriched ZBTB48 truncations pulled down by circRNF10 probe in SYNS03. **i** qPCR assays of the mRNA expression of ZBTB48 in circRNF10-overexpressed SYNS05 and SYNS06. **j** Western blotting showed the protein level change of ZBTB48 after circRNF10 overexpression in SYNS05 and SYNS06. **k**, **l** The circRNF10-silenced SYNS03 (**k**) or overexpressed SYNS05 (**l**) were treated with CHX (50 μg/ml) and the expression of ZBTB48 protein was detected by western blotting and the half-life time (t_1/2_) was quantitative analysis. **m**, **n** The circRNF10-silenced SYNS03 (**m**) or overexpressed SYNS05 (**n**) were treated with or without MG-132 (50 μM) for 6 h, and ZBTB48 expression was detected by western blotting. **o**, **p** Ubiquitination assays showed the ZBTB48 ubiquitination levels in GSCs followed by circRNF10 silencing (**o**) or overexpressed (**p**). The GSCs were pretreated with MG132 (50 μM) for 6 h. All data are expressed as the mean ± SD (three independent experiments). *p < 0.05; **p < 0.01; ***p < 0.001; ns, no significance

To explore the specific mechanism by which circRNF10 assists GSCs in evading ferroptosis, we used RNA-pulldown assay to detect proteins bound by the circRNF10 probe and hereafter performed mass spectrometry analysis of the interacting proteins to obtain a list of proteins that may bind to circRNF10 (Table S6). Meanwhile, we predicted proteins that interact with circRNF10 by the aid of CatRAPID (http://service.tartaglialab.com/page/catrapid_group) (Table S7). Considering that transcription factors are important mediators in the regulation of ferroptosis and malignant tumor signaling pathways, we conducted an intersection analysis of the CatRAPID prediction results, mass spectrometry analysis results, and all transcription factors from Cistrome Data Broswer (http://cistrome.org/db/#/stat) (Table S8), and determined that ZBTB48 was the only transcription factor that was most likely to bind to circRNF10 (Fig. 3a, b, Fig. S6a). To further confirm these findings, we performed co-localization experiments between circRNF10 and ZBTB48. As expected, circRNF10 was primarily located in the cytoplasm of GSCs, while ZBTB48 was distributed throughout different parts of GSCs, and both were co-localized in the cytoplasm (Fig. 3c). Then, to validate the interaction between circRNF10 and ZBTB48, RNA-pulldown and RIP assays were performed in SYNS03 and SYNS05. We observed that the wild-type biotinylated circRNF10 probe pulled down ZBTB48 while the mutant-type probe did not (Fig. 3d, Fig. S6b). The results of the RIP assay revealed that anti-ZBTB48 treatment detected more enriched circRNF10 than IgG. Moreover, compared with the negative control group, less accumulated circRNF10 was detected in anti-ZBTB48-treated SYNS03 and SYNS08 with circRNF10 knockdown (Fig. 3e, Fig. S6c). Similarly, more abundant circRNF10 was detected in SYNS05 and SYNS06 with circRNF10 overexpression (Fig. S6d, e). Additionally, we performed separate RIP assays for the nucleus and cytoplasm of GSCs, and qPCR results showed that circRNF10 was significantly enriched and amplified in the cytoplasmic fraction, whereas the binding between ZBTB48 and circRNF10 was almost undetectable in the nucleus (Fig. S6f, g). Furthermore, to investigate the precise location of the interaction between circRNRF10 and ZBTB48, on the basis of the prediction results of CatRAPID (Fig. 3f, upper panel), a series of Flag-tagged ZBTB48 truncation mutants were designed for RIP and RNA-pulldown assays (Fig. 3f, lower panel). The band of the RIP assay demonstrated that only the △428-688aa truncation mutant failed to enrich circRNF10 in anti-Flag treatment, while the other truncation mutants, △1-77aa, △1-152aa, and △1-427aa, enrich circRNF10 to varying degrees compared to IgG treatment (Fig. 3g). The RNA-pulldown assay showed that the biotinylated circRNF10 probe pulled down the △1-77aa, △1-152aa, and △1-427aa truncation mutants of ZBTB48, while the △428-688aa truncation mutant did not be pulled down by the RNA probe (Fig. 3h). Altogether, these conclusions clearly confirmed the N-terminal domain (1-427aa) of ZBTB48 is essential for its interaction with circRNF10.

### CircRNF10 inhibits ZBTB48 degradation via the ubiquitin‑proteasomal pathway

Having verified the molecular site of interaction, we investigated the specific regulatory mechanism of circRNF10 on ZBTB48 expression in GSCs. Firstly, qPCR was used to detect the expression level of ZBTB48 mRNA in GSCs with circRNF10 overexpression or knockdown. CircRNF10 overexpression had almost no effect on the expression level of ZBTB48 mRNA in SYNS05 and SYNS06(Fig. 3i). Similarly, there was no change in the ZBTB48 mRNA expression detected in SYNS03 and SYNS08 with circRNF10 knockdown (Fig. S6h). However, conspicuously changes in ZBTB48 expression at the protein level were detected by western blotting after gene manipulation of circRNF10 (Fig. 3j, Fig. S6i). These findings suggest that circRNF10 regulates the expression of ZBTB48 at the protein level rather than the transcriptional level. As validated by the CHX pulse-chase assay, the half-life of ZBTB48 was significantly shortened in circRNF10-silenced SYNS03(Fig. 3k). Correspondingly, overexpressing circRNF10 can obviously prolong the half-life of ZBTB48 (Fig. 3l). In addition, adding proteasome inhibitor MG132 can rescue the degradation of ZBTB48 caused by circRNF10 knockdown in SYNS03 (Fig. 3m). Meanwhile, MG132 treatment elicited a further accumulation of ZBTB48 on an already increased basis imposed by circRNF10 overexpression in SYNS05 (Fig. 3n). The above evidence supports that the binding of circRNF10 to ZBTB48 increases its stability, thereby leading to sustained high expression at the protein level. We speculate that circRNF10 may regulate the expression of ZBTB48 by affecting its post-translational modification. Ubiquitination is a common protein modification that is closely related to protein degradation. Most functional proteins in cells are degraded through the ubiquitin–proteasome system [25]. Therefore, we propose a hypothesis that circRNF10 maintains high expression in GSCs by reducing the ubiquitination of ZBTB48 as well as increasing its stability. To validate this conjecture, ubiquitination analysis of ZBTB48 was performed, and the experimental results showed that circRNF10 overexpression can significantly reduce the ubiquitination modification of ZBTB48 (Fig. 3o), while circRNF10 silencing is accompanied by an increase of the ubiquitination level of ZBTB48 (Fig. 3p). Taken together, the ubiquitination-dependent degradation of ZBTB48 can be disrupted by circRNF10, which binds to the N-terminal domain of ZBTB48 to increase its stability.

### CircRNF10 enhances ZBTB48 stability by competitively binding to MKRN3

**Fig. 4:**
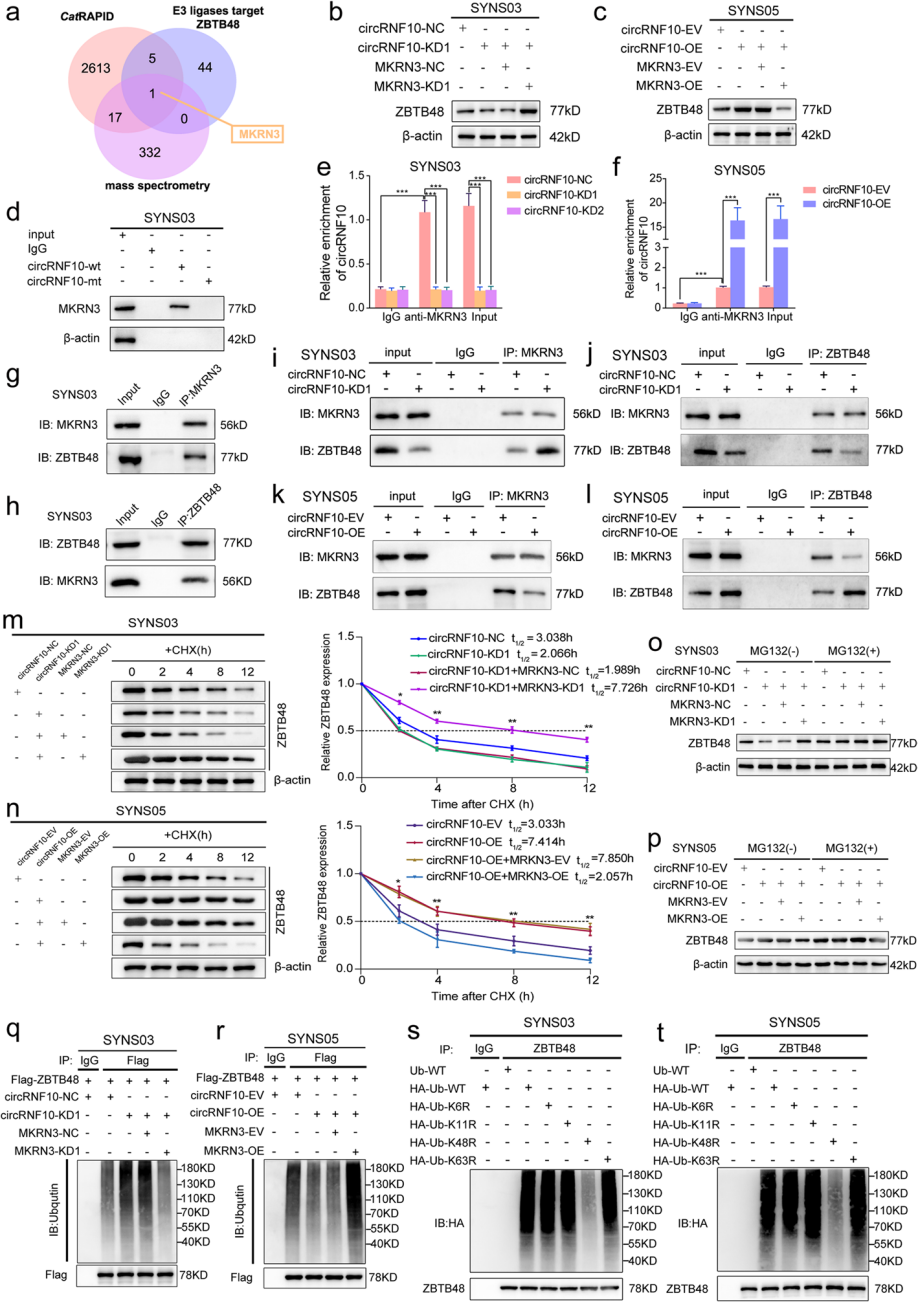
CircRNF10 interacts with ZBTB48 and prevents from degradation via inhibiting the ubiquitin- proteasomal pathway. **a** Venn diagram showed integrated CatRAPID, mass spectrometry and UbiBrowser results. **b**, **c** Western blotting for ZBTB48 in SYNS03 with circRNF10 knockdown followed by MKRN3 silencing (**b**) and circRNF10 overexpression with MKRN3 upregulation in SYNS05 (**c**). **d** RNA pulldown assays revealed the enriched MKRN3 pulled down by circRNF10 probe in SYNS03. **e**, **f** RIP assays displayed anti-MKRN3 treatment caused circRNF10 enrichment in circRNF10-silenced SYNS03 (**e**) and circRNF10-overexpressed SYNS05 (**f**). **g**, **h** Co-IP assays showed the interaction of ZBTB48 and MKRN3 in SYNS03. **i**-**l** Co-IP assays illustrated interaction efficiency of ZBTB48 and MKRN3 under the condition of circRNF10 knockdown in SYNS03 (**i**. **j**) and circRNF10 overexpression in SYNS05 (**k**, **l**). **m**, **n** Western blotting showed ZBTB48 expression in circRNF10-silenced combined with MKRN3-knockdown SYNS03 (**m**) or circRNF10 and MKRN3 co-overexpressed SYNS05 (l) by treated with CHX (50 μg/ml) and the half-life time (t_1/2_) of ZBTB48 protein was quantitative analysis. **o**, **p**. CircRNF10-silenced combined with MKRN3-knockdown SYNS03 (**o**) or circRNF10 and MKRN3 co-overexpressed SYNS05 (**p**) were treated with or without MG-132 (50 μM) for 6 h, and ZBTB48 expression was determined by western blotting. **q**, **r** In vivo ubiquitination assays showed the ZBTB48 ubiquitination levels followed by circRNF10-silenced combined with MKRN3-knockdown SYNS03 (**q**) or circRNF10 and MKRN3 co-overexpressed SYNS05 (**r**). **s**, **t** In vivo ubiquitination assays of polyubiquitin chains of ZBTB48 in SYNS03 (**s**) and SYNS05 (**t**) transfected with wild-type, K6R, K11R, K48R or K63R mutant ubiquitin plasmids. Data are presented as mean ± SD (three independent experiments). *p < 0.05; ** *P* < 0.01; ***P < 0.001; ns, no significance

Considering the E3 ubiquitin ligase mainly adds ubiquitin tags to protein substrates in the ubiquitination reaction, its enzymatic activity is crucial in the ubiquitination process [26]. Therefore, whether the ubiquitination level of ZBTB48 is affected by the activity of E3 ubiquitin ligase mediated by circRNF10 remains to be further explored. Firstly, we predicted the E3 ubiquitin ligases targeting ZBTB48 using UbiBrowser 2.0 (http://ubibrowser.bio-it.cn/ubibrowser_v3/home/index) (Table S9). Meanwhile, further screening was performed based on previous CatRAPID initial prediction results of proteins binding with circRNF10 and mass spectrometry analysis results obtained after RNA-pulldown. Notably, makorin ring finger protein 3 (MKRN3) might be the only E3 ubiquitin ligase that could bind to circRNF10 and target ZBTB48 as its substrate (Fig. 4a). To verify whether MKRN3 is involved in regulating the ubiquitination effect of circRNF10 on ZBTB48, western blot was conducted. The results revealed that the down-regulated expression of ZBTB48 caused by the silencing of circRNF10 could be rescued by the knockdown of MKRN3 (Fig. 4b). On the other hand, the increased ZBTB48 expression mediated by the up-regulated circRNF10 was reversed by the overexpression of MKRN3 (Fig. 4c). In addition, RNA pulldown and RIP assays were performed to confirm the interaction between circRNF10 and MKRN3 in SYNS03 and SYNS05 (Fig. 4d-f, Fig. S7a, d, e). Meanwhile, Co-IP assay results showed that MKRN3 could interact with ZBTB48 in GSCs (Fig. 4g, h, Fig. S7b, c). Furthermore, co-IP analysis was conducted under conditions of circRNF10 silencing or overexpression to explore whether circRNF10 has a competitive binding effect on MKRN3, leading to increased expression of ZBTB48. We observed circRNF10 silencing enhanced the binding function of MKRN3 to ZBTB48 while the expression level of ZBTB48 decreased (Fig. 4i, j). On the contrary, when circRNF10 was overexpressed, the interaction between MKRN3 and ZBTB48 was disrupted, accompanied by the elevated ZBTB48 expression (Fig. 4k, l). We then explored whether circRNF10 is necessary for MKRN3's ubiquitin ligase activity on ZBTB48. CHX pulse-chase assay showed that the circRNF10 downregulation-induced shortening of ZBTB48 half-life could be delayed by MKRN3 knockdown in SYNS03 (Fig. 4m). Correspondingly, the circRNF10 overexpression-induced prolongation of ZBTB48 half-life could be reduced by upregulation of MKRN3 in SYNS05 (Fig. 4n). In addition, MKRN3 silencing can rescue the degradation of ZBTB48 caused by circRNF10 knockdown in SYNS03, and that the high expression level of ZBTB48 tended to be stabilized with treatment of MG132 (Fig. 4o). Meanwhile, MKRN3 upregulation elicited a further accumulation of ZBTB48 on an already increased basis imposed by circRNF10 overexpression in SYNS05, as well as function of MKRN3 could be completely blocked by MG132 (Fig. 4p). Furthermore, ubiquitination assays revealed that the aggregation of ZBTB48-linked polyubiquitin chains caused by circRNF10 silencing could be abolished by MKRN3 deficiency (Fig. 4q). In contrast, replenishment of MKRN3 distinctly intensified the accumulation of polyubiquitinated ZBTB48 (Fig. 4r). Collectively, circRNF10 can competitively bind to MKRN3 and block its ubiquitin ligase activity, thereby improving the stability of ZBTB48 in GBM.

### MKRN3 catalyzes ZBTB48 ubiquitination depending on assembly K48-linked polyubiquitin chains

As a member of the RING family E3 ubiquitin ligase, MKRN3 can assemble atypical polyubiquitin chains to accomplish different atypical polyubiquitination modifications, which determine the downstream fate of proteins [27]. Atypical ubiquitination chains are usually assembled through K6, K11, K48, and K63-linked ubiquitin and considered to be the most prevalent and highly relevant to tumor development and protein degradation [28]. In validation assays, we constructed vectors carrying point mutations at Lys 6, 11, 48, and 63 (K6R, K11R, K48R, and K63R) respectively and bearing HA tags. Ubiquitination assays suggested that in GSCs, K48R strikingly reduced the accumulation of ZBTB48-linked polyubiquitin chains, while other ubiquitin point mutations did not have a diminishing effect on ZBTB48 polyubiquitination (Fig. 4r, s). However, the potential involvement of the other three types of K27, K29, and K33 ubiquitin chains in the post-translational modification of ZBTB48 and its impact on its function. To address this, we conducted additional investigations into the ubiquitination of ZBTB48 by K27, K29, and K33 chains. The results demonstrated that these three ubiquitin chains were not involved in the ubiquitination modification of ZBTB48 (Fig. S7f). Thus, the ubiquitinated degradation of ZBTB48 mainly depends on K48-linked polyubiquitin chains catalyzed and assembled by MKRN3.

### ZBTB48 is upregulated and confers ferroptosis resistance in glioma

**Fig. 5:**
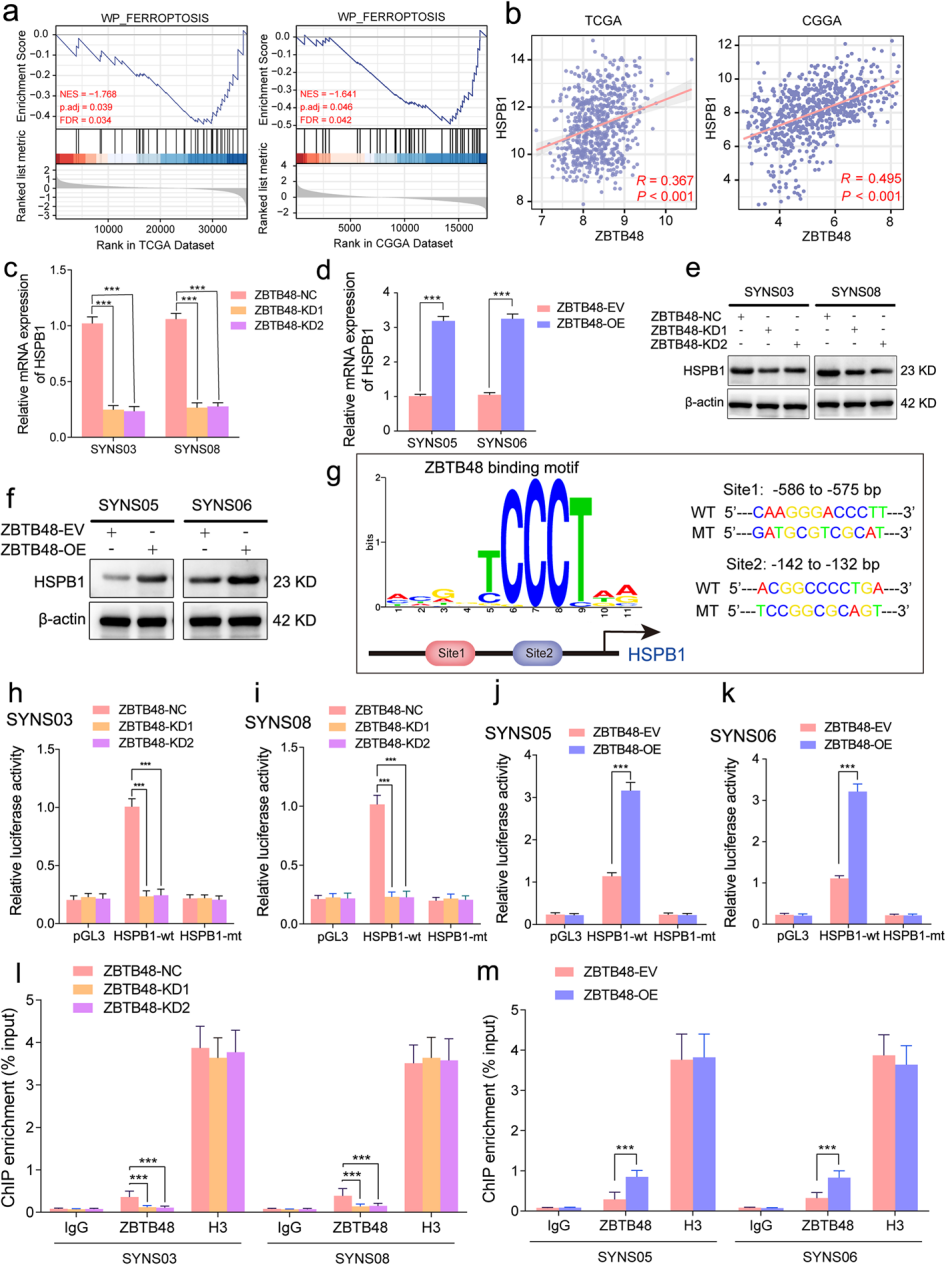
ZBTB48 transcriptionally upregulates HSPB1 expression in GSCs. **a** Correlation analysis of HSPB1 and ZBTB48 based on TCGA and CGGA datasets. **b** Differentially expressed genes obtained from TCGA and CGGA datasets divided into the low expression group and high expression group based on the median expression of ZBTB48 and GSEA plots depicting indicated ferroptosis signaling pathway in low expression group. **c**-**f**, qPCR assays of the transcriptional levels of HSPB1 in ZBTB48-silenced SYNS03 and SYNS08(**c**) as well as ZBTB48-overexpressed SYNS05 and SYNS06 (**d**). Western blotting showed the protein level change of HSPB1 after ZBTB48 knockdown (**e**) or overexpression (**f**) in GSCs. **g** Left schematic represents ZBTB48 binding motif obtained from AnimalTFDB database. Right schematic diagram shows the two putative ZBTB48 binding sites and matched mutant sequences in the HSPB1 promoter region for Dual-luciferase reporter assays. **h**–**k** The Dual-luciferase reporter assays revealed the luciferase promoter activities of HSPB1 with ZBTB48 scliencing in SYNS03 (**h**) and SYNS08 (**i**) or ZBTB48 overexpression in SYNS05 (**j**) and SYNS06 (**k**). **l**, **m** The ChIP qPCR showed that anti-ZBTB48 treatment could detect the enrichment difference of HSPB1 promoter sequence in ZBTB48-silenced SYNS03 and SYNS08 (**l**) or in ZBTB48-overexpressed SYNS05 and SYNS06 (**m**). All data are expressed as the mean ± SD (three independent experiments). *p < 0.05; **p < 0.01; ***p < 0.001; ns, no significance

Although our study has clarified the ubiquitination-mediated regulation of ZBTB48 by circRNF10, the biological function of ZBTB48 in glioma has not been reported. Primarily, we assessed the expression levels of ZBTB48 in CGGA and TCGA-glioma databases, and the obtained data indicated that the expression level of ZBTB48 elevated accompanied with the ascending grade of glioma, and its high expression was also observed in IDH1 wild-type and 1p19q non-codeletion groups (Fig. S8a-h). Furthermore, Kaplan–Meier survival analysis illustrated patients with high ZBTB48 expression had shorter survival time compared to low expression (Fig. S8i, j). These bioinformatics analysis results signified that ZBTB48 upregulation is related to poor prognosis in glioma patients. Besides, we further performed GSEA of the relationship between ZBTB48 and ferroptosis based on the TCGA-glioma and CGGA datasets. According to the analysis, the WP_FERROPTOSIS pathway was enriched in the low ZBTB48 expression groups (Fig. 5a). Consequently, the supervisory capacity of ZBTB48 for ferroptosis in GBM was validated by a series of experimental studies. The results showed that overexpression of ZBTB48 in SYNS05 and SYNS06 noticeably decreased the intracellular content of MDA and upregulated the level of GSH (Fig. S8k, l). In addition, the abundance of the reduced probe R-BODIPY was appreciably expanded estimating by the BODIPY (581/591) C11 probe in ZBTB48-overexpressed GSCs (Fig. S8m, n). Therefore, ZBTB48 is a crucial regulatory factor that mediates the ferroptosis resistance of GSCs.

### Upregulation of ZBTB48 invades ferroptosis mediated by circRNF10-silenced in GSCs

Since the discovered mutual interaction relationship between circRNF10 and ZBTB48 and their oncogenic and ferroptosis-defending role in glioma, we further evaluated whether ZBTB48 can serve as a pivotal downstream factor for circRNF10 against ferroptosis in GBM. The increase of MDA level and the decrease of GSH content caused by circRNF10 knockdown can both be restored by overexpression of ZBTB48 (Fig. S9a, b). The BODIPY (581/ 591) C11 assays indicated an obviously increase in lipid peroxidation after circRNF10 silencing in SYNS03 and SYNS08, while ZBTB48 overexpression dropped the antioxidant capability of in SYNS03 and SYNS08 in SYNS03 and SYNS08 (Fig. S9c, d). Moreover, we also explored the effect of ZBTB48 overexpression combined with circRNF10 knockdown on cell viability and proliferation of GSCs. MTS assay illustrated that the decreased cell viability of SYNS03 and SYNS05 after circRNF10 knockdown could be notably increased by ZBTB48 overexpression (Fig. S9e, f). NSFA and ELDA showed that ZBTB48 overexpression could restore the diminished stemness maintenance and proliferation ability of circRNF10 silenced SYNS03 and SYNS05 (Fig. S9g-j). In addition, EdU assay was also performed. Similarly, ZBTB48 overexpression could visibly enhance the proliferation capacity of SYNS03 and SYNS05 impaired by circRNF10 knockdown (Fig. S9k, l). The cell cycle assays also indicated that overexpressed ZBTB48 could facilitate the G1/S phase transition of circRNF10-silenced SYNS03 and SYNS05 (Fig. S9m). These data indicated that circRNF10-silencing induced ferroptosis could be rescued by ZBTB48 overexpression in GSCs.

### ZBTB48 is a transcriptional regulator of HSPB1 expression in GSCs

As there is no reported research on the regulatory function of ZBTB48 in ferroptosis, we conducted correlation analysis between ZBTB48 and ferroptosis-related genes in the CGGA and TCGA-glioma databases. The correlational analyses revealed that only one candidate gene, heat shock protein family B (small) member 1 (HSPB1), was positively correlated with ZBTB48 (R > 0.3, *p* < 0.05) in the transcriptional level (Fig. 5b), and HSPB1 has been reported in previous studies to act as a resistance factor mediating cell evade from ferroptosis [29]. To identify whether ZBTB48 can regulate the expression of HSPB1, qPCR and western blotting assay were performed under ZBTB48 knockdown or overexpression conditions in GSCs. We observed that ZBTB48 knockdown led to an evident decrease in both HSPB1 mRNA and protein levels (Fig. 5c-e), while ZBTB48 overexpression upregulated the expression of HSPB1 (Fig. 5d-f).

Given that ZBTB48 has transcription factor function, we further investigated whether ZBTB48 could transcriptionally regulate HSPB1. The complete coding sequence region of HSPB1 was excavated by NCBI, and nucleotides including the HSPB1 promoter (-2000 to 100 of the human HSPB1 locus) were used to predict potential transcription factor binding sites. Potential binding sites for transcription factors to the HSPB1 promoter region were predicted via the AnimalTFDB database (http://bioinfo.life.hust.edu.cn/AnimalTHDB4/#/) (Table S10). Subsequently, we analyzed the potential ZBTB48 transcriptional binding sites in the HSPB1 promoter sequence (Fig. 5g, left panel). Furthermore, two mutated nucleotide fragments regarding the binding sites of ZBTB48 were designed within the promoter region of HSPB1 (Fig. 5g, right panel). The results of the luciferase reporter gene assay revealed that ZBTB48 knockdown observably decreased the luciferase activity of pGL3-HSPB1-wt in SYNS03 and SYNS08 (Fig. 5h, i), while ZBTB48 overexpression enhanced the luciferase activity of pGL3-HSPB1-wt in SYNS05 and SYNS06 (Fig. 5j, k). In addition, the ChIP assay also displayed that ZBTB48 silencing decreased the enrichment of HSPB1 motivated by anti-ZBTB48 treatment in SYNS03 and SYNS08, while ZBTB48 treatment signally increased the enrichment of HSPB1 through ZBTB upregulation in SYNS05 and SYNS06 (Fig. 5l, m). Taken together, ZBTB48 has a transcriptional regulatory function for the expression of HSPB1 in GSCs.

### HSPB1 remolds iron metabolism to facilitate the escape of GSCs from ferroptosis

**Fig. 6:**
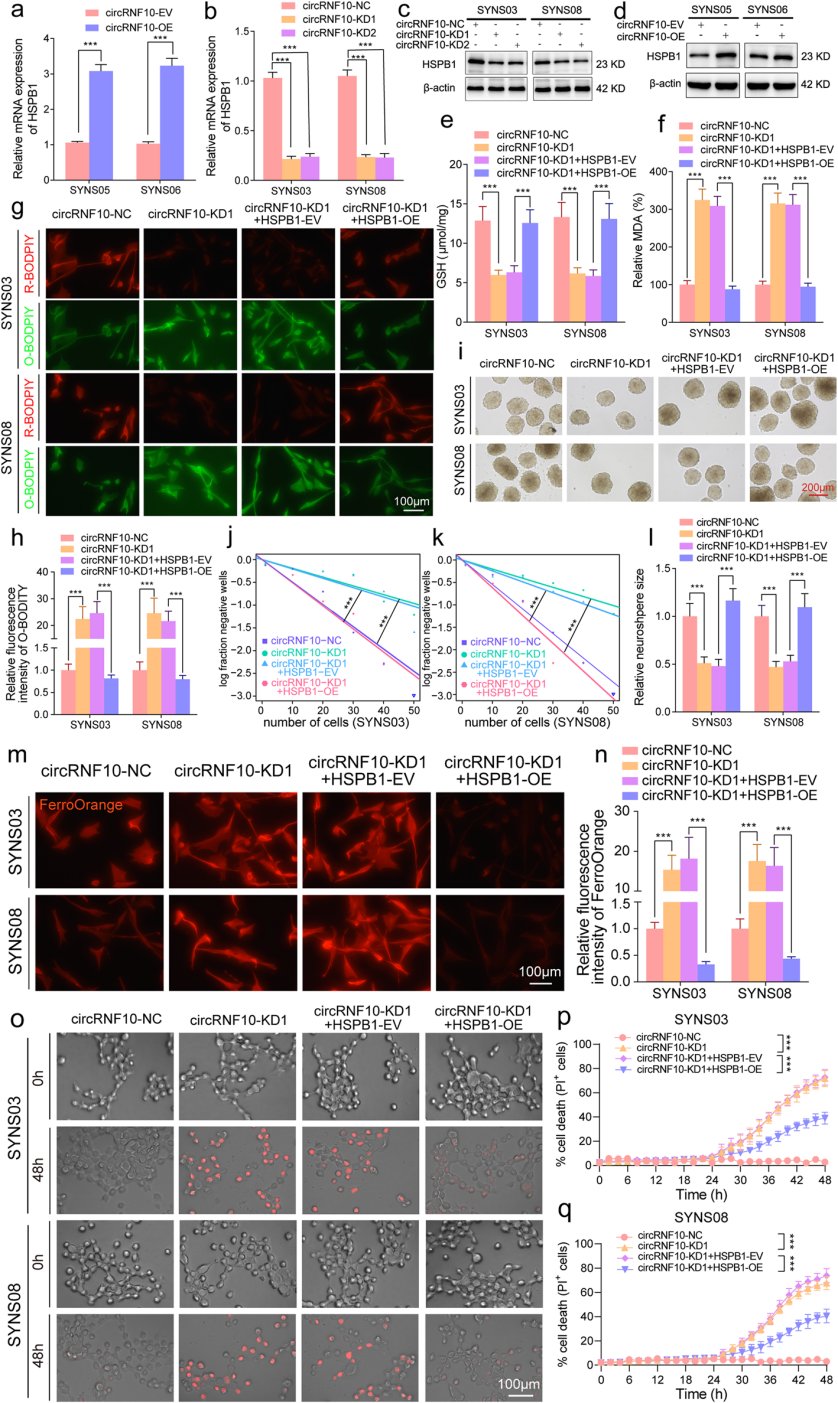
CircRNF10 defends ferroptosis via upregulating HSPB1 in GSCs. **a**, **b** qPCR assays of the transcriptional levels of HSPB1 in circRNF10-overexpressed SYNS05 and SYNS06 (**a**) as well as circRNF10-knockdown SYNS03 and SYNS08 (**b**). **c**, **d** Western blotting showed the protein level change of HSPB1 after circRNF10 knockdown (**c**) or overexpression (**d**) in GSCs. **e**, **f**. GSH (e) and MDA (**f**) contents measured in SYNS03 and SYNS08 with circRNF10 knockdown followed by HSPB1 overexpression. **g**, **h** Lipid peroxidation levels (**g**) detected by BODIPY (581/591) C11 probe in circRNF10-knockdown SYNS03 and SYNS08 followed by HSPB1 upregulation and the relative fluorescence intensity of O-BODIPY quantified by Image J (**h**). Scale bar = 100 μm. **i**, Representative images of NSFA with circRNF10 knockdown in SYNS03 and SYNS08 followed by HSPB1 overexpression. Scale bar = 200 μm. **j**, **k** ELDA in SYNS03 (**j**) and SYNS08 (**k**) after circRNF10 silencing followed by HSPB1 overexpression. **l** Relative sizes of neurospheres of circRNF10-silenced SYNS03 and SYNS08 after HSPB1 upregulation. **m**, **n** Representative images of FerroOrange staining assays illustrated the intracellular Fe^2+^ accumulation of circRNF10-knockdown SYNS03 and SYNS08, followed by HSPB1 overexpression (**m**). The relative fluorescence intensity of FerroOrange probe quantified by Image J (**n**). Scale bar = 100 μm. **o** Representative images of cell death in SYNS03 and SYNS08 after 48 h of indicated treatments. Scale bar = 100 μm. **p**, **q** Real-time analysis of cell death in SYNS03 (**p**) and SYNS08 (**q**) with circRNF10 knockdown followed by HSPB1 upregulation. All data are expressed as the mean ± SD (three independent experiments). *p < 0.05; **p < 0.01; ***p < 0.001; ns, no significance

To our knowledge, role for HSPB1 in iron metabolism has been previously reported [30]. Erastin treatment can induce the activation of HSPB1 to trigger the ferroptosis resistance mechanism in tumor cells. Meanwhile, protein kinase C can mediate the phosphorylation of HSPB1 and reduce iron-mediated ROS production to defend ferroptosis [31]. Therefore, it is worth to reveal whether circRNF10 can protect GSCs from ferroptosis and accelerate GBM progression by regulating the expression of HSPB1. Firstly, qPCR and western blotting assays showed that circRNF10 positively regulates HSPB1 at both protein expression and transcription levels (Fig. 6a-d). Functionally, silencing of circRNF10 resulted in a decrease of cell viability, which could be reversed by the upregulation of HSPB1 in SYNS03 and SYNS08 (Fig. S10a, b). Additionally, the combined regulation of circRNF10 and HSPB1 on ferroptosis in GSCs was explored, and the results demonstrated that the decrease of intracellular GSH levels and the increase of MDA levels caused by circRNF10 silencing could be rescued by HSPB1 overexpression. Moreover, accumulated lipid peroxidation caused by circRNF10 knockout in GSCs could be visibly eliminated by HSPB1 upregulation as detected by the BODIPY (581/591) C11 assay (Fig. 6e-h).

In terms of the proliferation and stemness maintenance abilities of GSCs, as evidenced by NSFA, ELDA, and EdU assays, the diminishing effects involved in circRNF10 silencing can all be restored by the overexpression of HSPB1(Fig. 6i-l, Fig. S10c, d). Furthermore, FerroOrange probe was used to provide feedback on the remodeling effect of HSPB1 on iron metabolism in GSCs. As expected, the accumulation of labile ferrous (Fe^2+^) ions caused by circRNF10 knockout can be completely eliminated by HSPB1 overexpression (Fig. 6m, n). Finally, the HSPB1 overexpression showed dynamic relief of the kinetics of cell death triggered by knockdown of circRNF10 in GSCs (Fig. 6o-q). In summary, downregulation of HSPB1 mediated by circRNF10 silencing is sufficient to remodel iron metabolism pool of the GSCs, rendering GBM susceptible to ferroptosis.

### IGF2BP3 strengthens stability of circRNF10 in GSCs

**Fig. 7:**
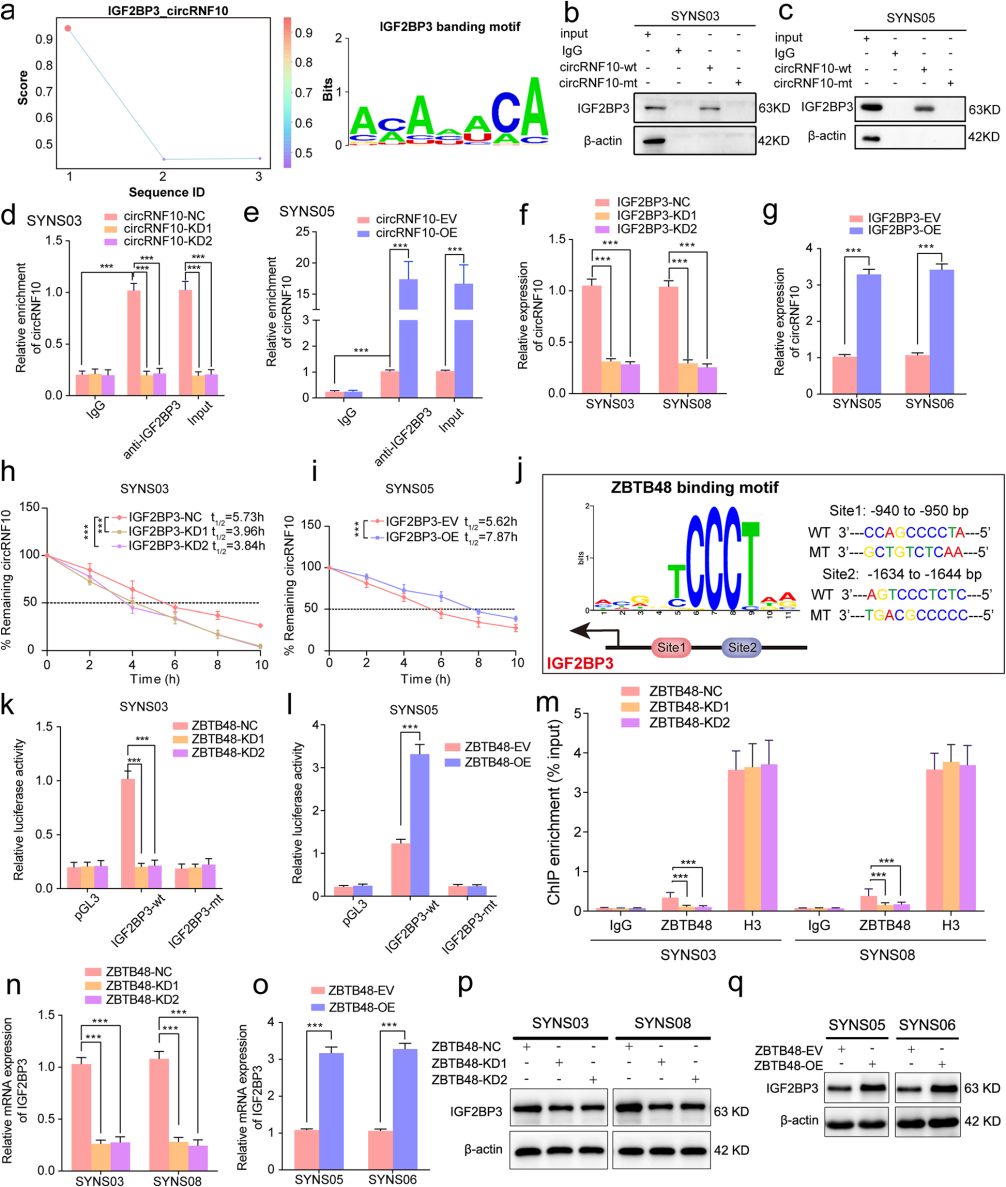
IGF2BP3 maintain the stability of circRNF10 and is transcriptionally upregulated by ZBTB48 in GSCs. **a** IGF2BP3 was predicted by RBP suite to interact with circRNF10. **b**, **c** RNA pull-down assays showing the biotinylated circRNF10 probes pull-down IGF2BP3 protein in SYNS03 (**b**) and SYNS05 (**c**). **d**, **e** RIP assays showing anti-IGF2BP3 treatment enriched with circRNF10 after circRNF10 overexpression (**d**) or knockdown (**e**). **f**, **g** qPCR showing circRNF10 expression after knockdown (**f**) or overexpression (**g**) of IGF2BP3 in GSCs. **h**, **i** RNA stability assays showing the half-life of circDNF10 in IGF2BP3-silenced (**h**) or overexpressed (**i**) GSCs followed by actinomycin D treatment. **j** Left schematic represents ZBTB48 binding motif and promoter region of IGF2BP3. Right schematic diagram shows the two putative binding sites and matched mutant sequences in the promoter region for Dual-luciferase reporter assays. **k**, **l** The Dual-luciferase reporter assays revealed the luciferase promoter activities of IGF2BP3 with ZBTB48 silencing in SYNS03 (**k**) and ZBTB48 overexpression in SYNS05 (**l**). **m** The ChIP qPCR showing the enrichment difference of IGF2BP3 promoter sequence via anti-ZBTB48 treatment in ZBTB48-silenced SYNS03 and SYNS08. **n**-**q** qPCR and Western blotting assays displayed the expression level’ s change of IGF2BP3 in ZBTB48-silenced (**n**, **o**) and ZBTB48-overexpressed GSCs (**p**, **q**). All data are expressed as the mean ± SD (three independent experiments). *p < 0.05; **p < 0.01; ***p < 0.001; ns, no significance

As RNA binding proteins (RBPs) are involved in various processes of circRNAs, such as splicing, synthesis, stabilization, and degradation, we used the RBP suite database (http://www.csbio.sjtu.edu.cn/bioinf/RBPsuite/) to predict and score the RBPs that may bind to circRNF10 (Table S11). Among the predicted and evaluated RBPs, insulin like growth factor 2 mRNA binding protein 3 (IGF2BP3) obtained the highest score, indicating the highest credibility of its interaction with circRNF10. Meanwhile, the binding segment and motif between circRNF10 and IGF2BP3 were obtained through the RBP suite analysis (Fig. 7a). To further confirm the interaction between circRNF10 and IGF2BP3, RNA-pulldown and RIP assays were performed in SYNS03 and SYNS05. Subsequent analysis revealed that the circRNF10 probe exhibited an affinity for IGF2BP3 protein, and anti-IGF2BP3 treatment resulted in the enrichment of circRNF10 (Fig. 7b-e, Fig. S11a, b). As noted, IGF2BP3 contributes to the execution of RNA stability. Therefore, we posited that IGF2BP3 combines with and protects circRNF10 from degradation. To begin testing this hypothesis, we disrupted IGF2BP3 expression and detected circRNF10 levels by qPCR assay. Decreased circRNF10 expression was observed in SYNS03 and SYNS08 (Fig. 7h, Fig. S11d). Correspondingly, IGF2BP3 overexpression led to a complete reverse of circRNF10 levels in SYNS05 and SYNS06 (Fig. 7i, Fig. S11c). Consistent with this hypothesis, the determination of circRNF10's half-life after actinomycin D treatment showed that IGF2BP3 disruption accelerated circRNF10's decay in SYNS03, whereas overexpression of IGF2BP3 delayed the attenuation time of circRNF10 in SYNS05. In sum, IGF2BP3 enhances the stability of circRNF10 by a mechanism of RNA–protein interaction in GSCs.

### ZBTB48 transcriptionally upregulates IGF2BP3 and triggers a positive feedback loop in GSCs

We sought to better understand how IGF2BP3 selectively strengthen and maintain the circRNF10 stability in GSCs. Therefore, the potential transcriptional regulatory role of ZBTB48 on IGF2BP3 was further investigated. Intriguingly, the analysis and prediction consequence from the AnimalTFDB database indeed comprised possible ZBTB48 binding sites in the promoter region of IGF2BP3 (Fig. 7j left panel, Table S12), then nucleic acid sequences carrying point mutations in the promoter regions were designed and cloned into pGL3 plasmids (Fig. 7j right panel). Knockdown of ZBTB48 obviously reduced luciferase activity of pGL3-IGF2BP3-wt compared to the negative control in SYNS03 and SYNS08 (Fig. 7k, Fig. S11e). By contrast, ZBTB48 overexpression showed a significant enhancement in luciferase activity in SYNS05 and SYNS06 (Fig. 7l, Fig. S11f). Likewise, silencing of ZBTB48 generated declined enrichment of IGF2BP3 by anti-ZBTB48 treatment in ChIP assay, while the contrary findings were obtained via ZBTB48 upregulation (Fig. 7m, Fig. S11g). Correspondingly, we observed that addback of ZBTB48 expression could completely expand IGF2BP3 expression in both transcriptional and protein levels while decrease after ZBTB48 knockdown, as determined by measuring both qPCR and west blotting assays (Fig. 7n-q). In conclusion, these data underscored the ZBTB48 could transcriptionally upregulated IGF2BP3 and a positive feedback loop was composed by ZBTB48, IGF2BP3, and circRNF10 in GSCs to enhance tumorigenic efficacy.

### CircRNF10 regulates GBM tumor burden via circRNF10/ZBTB48/IGF2BP3 feedback loop in vivo

**Fig. 8:**
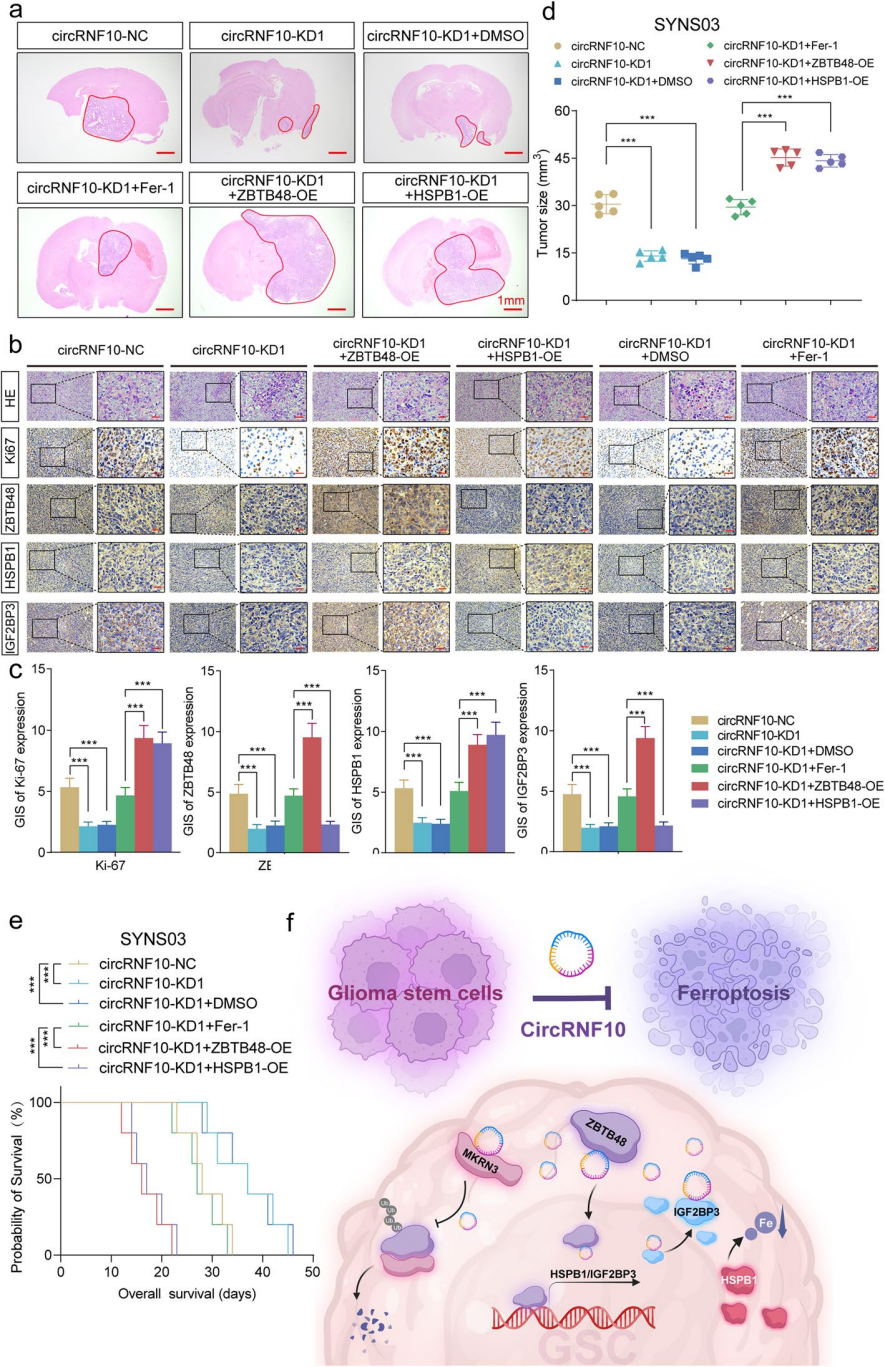
circRNF10/ZBTB48/IGF2BP3 feedback loop regulates GBM tumor burden in vivo. **a** Representative photographs showed the sizes of intracranial tumors in the coronal position of the circRNF10 knockdown group and combined with ZBTB48, HSPB1 overexpression and Fer-1 treatment groups. Scale bar = 1 mm. **b** Representative immunohistochemical images showed the staining and expression of ZBTB48, HSPB1, IGF2BP3 and Ki-67 of the multiple groups mentioned above. Scale bar = 50 μm. **c** The quantified staining results of Ki-67, ZBTB48, HSPB1and IGF2BP3 according to the German IHC scoring system (GIS). **d** The tumor sizes of different groups mentioned above. **e** Kaplan–Meier survival curves illustrates the survival times of NOD/SCID mice of different mentioned groups. **f** Schematic diagram showing circRNF10/ZBTB48/IGF2BP3 feedback loop promotes glioma tumorigenesis via HSPB1 mediated ferroptosis defending and MKRN3 inhibition. All data are expressed as the mean ± SD (three independent experiments). *p < 0.05; **p < 0.01; ***p < 0.001; ns, no significance

We next investigated whether circRNF10/ZBTB48/ IGF2BP3 feedback loop impacts GBM tumor burden in vivo. In agreement with our in vitro findings, SYNS03 with circRNF10 knockdown orthotopic xenograft tumor volumes were significantly lessened compared to the negative control. By contrast, Fer-1 treatment could partially attenuate the tumor suppressor effect of circRNF10 silencing, as evidenced by intracranial GBM tumor burden. In distinction, overexpression of ZBTB48 or HSPB1 can completely reverse the tumorigenic ability weakened by circRNF10 knockdown (Fig. 8 a, d). The positive feedback feature in GSCs mediated by the interaction among circRNF10, ZBTB48, with IGF2BP3 was morphologically identified by immunohistochemical staining of orthotopic xenograft tumor specimens. The ICH staining intensity of Ki-67, ZBTB48, IGF2BP3 and HSPB1 indicated that circRNF10 could regulate the expression of all other molecules in the feedback loop. However, HSPB1 is not involved in the feedback loop and can only affect the positive level of Ki67. (Fig. 8b, c). Kaplan–Meier survival analysis indicated that circRNF10 silencing significantly extended the survival of mice. In comparison, Fer-1 treatment group disrupted the survival benefit originating from circRNF10 knockdown, while the upregulation of ZBTB48 or HSPB1 completely abrogated the tumor suppressor effect mediated by circRNF10 knockdown, significantly reducing the survival time of mice (Fig. 8e). These data emphasize circRNF10 can establish a positive feedback loop with ZBTB48 and IGF2BP3 upregulating HSPB1 to remodel the iron metabolism of GSCs and resist ferroptosis. Meanwhile, circRNF10 can act as a competitive inhibitor of E3 ubiquitin ligase MKRN3, impeding its enzymatic activity and preventing ZBTB48 degradation. The amplification of oncogenic signaling and ferroptosis defense ultimately primes GBM progression (Fig. 8f).

## Discussion

Aberrant expression of circRNAs is a significant hallmark in various malignancies. Due to their high stability and tissue specificity, circRNAs are ideal biomarkers for therapeutic effects, and represent promising targets or adjuvants for anticancer therapy. The progress made in investigations in this research field provides essential support for the future development of evolutionary diagnostic, prognostic and treatment strategies for cancer therapy [32]. Meanwhile, multiple circRNAs have been reported to affect the progression of GBM through involvement in resistance to radio- and chemotherapy, angiogenesis, and regulation of the microenvironment [33]. For instance, circNCAPG is significantly upregulated in GBM tissue and activates the TGF-β pathway through the U2AF65/circNCAPG/RREB1 positive feedback loop to promote the malignant phenotype of GSCs [34]. Hypoxia-induced circADAMTS6 can accelerate GBM progression via the ANXA2/NF-κB pathway in a TDP43-dependent manner [35]. Additionally, tumor treating fields (TTF) -mediated downregulation of circMMD can inhibit glioma progression by the FUBP1/FIR/DVL1 and miR-15b-5p/FZD6 pathways [36]. Circular EZH2-encoded EZH-92aa mediates immune escape of GBM by suppressing NKG2D ligands [37]. Furthermore, our previous study showed that circLRFN5 inhibits the progression of glioblastoma through the PRRX2/GCH1-mediated ferroptosis [38]. In this study, we identified a novel circular RNA, circRNF10, which is significantly upregulated in GBM. Importantly, the expression of circRNF10 is positively correlated with glioma grade, and patients with high circRNF10 expression exhibit an unfavorable prognosis. Both in vitro and in vivo assays indicate that circRNF10 can promote the malignant progression of GBM by inhibiting ferroptosis in GSCs. In summary, our study provides precise insights into the regulatory mechanisms of circular RNAs in GBM ferroptosis, supplementing the rationale of circRNF10 as a prognostic indicator for glioma patients.

Ferroptosis is governed by the intricate interplay of iron metabolism, lipid peroxidation, and antioxidant systems, and any perturbation in these processes can incite ferroptosis. In response to lipid peroxidation, cells employ multiple defense mechanisms including the classical pathway mediated by GPX4, the non-classical pathway mediated by FSP1, and a crucial pathway involving the interaction of dihydroorotate dehydrogenase (DHODH) with GPX4 to reduce ubiquinone to form ubiquinol in the inner mitochondrial membrane [39]. A new study elucidated that lipid metabolic remodeling caused by CDKN2A deficiency mutation upregulates membrane LPO and consequently primes GBM for ferroptosis [40]. Epigenetically silenced lncRNA SNAI3-AS1 has been found to promote ferroptosis in glioma by disrupting the m^6^A-dependent recognition of Nrf2 mRNA through SND1 [41]. Apart from being regulated by lipid metabolism, the maintenance of cytoplasmic iron homeostasis also acts as a targetable vulnerability in ferroptosis. Recent research has indicated that FHOD1 overexpression in glioma attenuates ferroptosis by targeting HSPB1 signaling [42]. Additionally, HSPB1 and phosphorylated HSPB1 can reduce the iron uptake of cancer cells from the intracellular environment, thus maintaining iron homeostasis and reducing susceptibility to ferroptosis [43]. Existing research indicates that HSPB1 may act as a potential oncogene in glioblastomas and is associated with poor prognosis in patients [44, 45]. Our study demonstrates that circRNF10 can upregulate HSPB1 expression in GSCs by stabilizing ZBTB48, leading to ferroptosis escape and promoting progression in GBM. On the other hand, our research also elucidates the remodeling of iron metabolism in GBM as a possible therapeutic approach.

It has been reported that ZBTB48 prevents excessive elongation of telomeres by inducing the initiation of telomere trimming, thus maintaining the appropriate telomere length. The ZBTB48 gene is located in chromosome 1p36.12 region, which is one of the familiar oncogenic gene deletion regions associated with multiple human malignancy [46]. Therefore, ZBTB48 may act as a primary role in the pathogenesis of cancer. Previous researches have suggested that ZBTB48 has an auxo-action for progression colorectal cancer, and cervical cancer, and high expression of ZBTB48 is associated with poor prognosis in patients [47, 48]. In this study, bioinformatics exploration based on TCGA and CGGA databases revealed a significant upregulation of ZBTB48 expression in high-grade gliomas, while a significant enrichment of ferroptosis pathway was observed in the ZBTB48 low-expression group. Furthermore, we demonstrated through assays that ZBTB48 can enhance the tumorigenicity of GSCs by inhibiting ferroptosis. Mechanistically, the interaction between circRNF10 and the N-terminal domain of ZBTB48 can enhance protein stability. In addition, considering the specificity of the ZBTB48 gene locus, which involves the important molecular pathological feature of chromosomal 1p19q co-deletion in gliomas, we also observed a significant downregulation of ZBTB48 in the 1p19q co-deletion group. Thus, our findings provided a novel biomarker and target in terms of ferroptosis defending for GBM diagnostic prediction and clinical outcome evaluation.

Cancer cells require high-efficiency protein translation rates to maintain the speedy proliferation behavior. The maintenance of protein homeostasis is a major metabolic event mastering proliferation of tumor, but its precise regulatory mechanisms in tumors are not well interpreted [49]. The Ubiquitin–Proteasome System (UPS) is a multi-component system responsible for protein degradation within cells, which is involved in various cellular processes such as cellular growth and differentiation, DNA replication and repair, cellular metabolism, and immune response by regulating the degradation of most intracellular proteins in eukaryotic cells [50]. As far as malignant tumors are concerned, the UPS can affect the survival of tumor cells by promoting the degradation of tumor suppressor proteins such as p53 or blocking the degradation of oncogenic proteins [51]. The functions of a series of E3 ubiquitin ligases have been reported in glioma, for instance, radiation induces E3 ligase IRAK1 expression to promote radioresistance of glioma by decreasing the ubiquitination of PRDX1 and suppressing autophagy [52]. In contrast, E3 ligase TRIM56 is transcriptionally upregulated by SP1 and impacts IQGAP1-CDC42 signaling to facilitate glioma migration and invasion [53]. It can be indicated that E3 ligases could act as tumor suppressors or promote malignant progression in glioma. MKRN3 contains four zinc finger domains, including three C3H1 motifs and one C3H4 ring finger domain to participates in gene transcription and ubiquitination process. The MKRN3 protein Initial studies found that loss-of-function mutations of MKRN3 lead to human central precocious puberty [54]. In addition, as an E3 ubiquitin ligase, MKRN3 mutations are associated with the progression of osteosarcoma and non-small cell lung cancer [55, 56]. In this study, the tumor-suppressor function of MKRN3 in glioma was discovered depending on E3 ubiquitin ligase activity. Mechanistically, MKRN3 can initiate the ubiquitination and degradation of ZBTB48 to reduce its oncogenic effects. Moreover, circRNF10 can competitively bind with MKRN3 and indirectly regulate ZBTB48 protein expression by blocking its enzymatic activity. It is also demonstrated that the ubiquitinated regulation of protein homeostasis in GSCs contributes to ferroptosis.

With the increasing understanding of circRNAs, it has been proposed in numerous studies that circRNAs can interact with RBPs to regulate cancer progression [57]. For instance, circDCUN1D4 can bind to the HuR protein and acts as a scaffold to facilitate the interaction of HuR and TXNIP mRNA, thereby increasing the stability of TXNIP mRNA to delay lung adenocarcinoma progression [58]. In addition, circNSUN2 can bind to IGF2BP2 and HMGA2, forming the circNSUN2/IGF2BP2/HMGA2 RNA–protein ternary complex, which contributes to promoting colorectal carcinoma metastasis [59]. Moreover, the overexpression of HnRNP-L, a multifunctional RNA-binding protein, is involved in upregulating circCSPP1 to furtherly target EGR1 and lead to malignant development of prostate cancer [60]. In present study, we discovered that IGF2BP3 functions as a binding factor of circRNF10, resulting in increased stability and enhanced oncogenic activity of circRNF10. Strikingly, the transcriptional regulation of IGF2BP3 by ZBTB48 leads to the formation of a positive feedback loop that continuously activates and strengthens the tumor-initiating potential of GSCs.

The initiation and progression of malignant tumors is not involved in single-gene abnormalities, but rather a consequence of the accumulation of multiple gene unconventional performance acting in synergy. Previous study has shown that CircKPNB1 mediates a positive feedback loop and promotes the malignant phenotypes of GSCs via TNF-α/NF-κB signaling [61]. In this particular study, ZBTB48, acting as a transcription factor, was clarified to stimulate the overexpression of IGF2BP3, hence forming a positive feedback loop, which enabled the continuous and abnormal upregulation of circRNF10, ZBTB48, and IGF2BP3, and activated HSPB1-mediated ferroptosis defense mechanisms in GSCs. Importantly, our finding reveals that circRNF10/ZBTB48/IGF2BP3 feedback loop impedes ferroptosis and enhances the malignant properties of GSCs.

## Conclusion

In summary, we characterized circRNF10 as an oncogene to promote proliferation and stemness sustenance of GSCs through defending ferroptosis in GBM. Mechanistically, circRNF10 can enhance protein stability and transcriptional activity of ZBTB48 by directly binding, promoting upregulation of HSPB1 and IGF2BP3 expression, which facilitates the formation of a circRNF10/ZBTB48/IGF2BP3 positive feedback loop, and remodels iron metabolism in GSCs. Additionally, circRNF10 can competitively bind to MKRN3 and block E3 ubiquitin ligase activity, thereby reducing the ubiquitination and degradation of ZBTB48, further enhancing expression and amplifying oncogenic function of ZBTB48. Moreover, upregulation of circRNF10 predicts unfavorable prognosis in glioma patients. Notably, circRNF10 may serve as a biomarker for glioma patients and manipulating circRNF10 as a vulnerability to target ferroptosis would be a potential orientation for GBM molecular therapies.

### Supplementary Information

**Additional file 1: Fig. S1. **Validation of the expression of the top 10 upregulated circRNAs in glioma and normal brain tissues (NBT). qPCR displays the expression of top 10 circRNAs in glioma and NBT. All data are expressed as the mean ± SD (three independent experiments). *p < 0.05; **p < 0.01; ***p < 0.001; ns, no significance.

**Additional file 2: Fig. S2. **CircRNF10 overexpression promotes viability, proliferation, neurospheres formation and stemness of GSCs. a. qPCR showed the expression of circRNF10 in GSCs derived from GBM patients. b. qPCR assays displayed the expression of circRNF10 in SYNS05 and SYNS06 after circRNF10 overexpression. c, d. MTS assays showed the cell viabilities of circRNF10-upregulated SYNS05 (c) and SYNS06 (d). e, f. Representative images of EdU assays showed the proliferation of circRNF10-overexpressed SYNS05 and SYNS06. Scale bar = 100μm. g-j. Representative images of NSFA with circRNF10 overexpression in SYNS05 and SYNS06 (g). Relative sizes of neurospheres of circRNF10-upregulated SYNS05 and SYNS06 (h). Scale bar = 100μm. ELDA in SYNS05 (i) and SYNS06 (j) after circRNF10 overexpression. k. Western blotting showed the stemness markers expression in circRNF10-overexpressed SYNS05 and SYNS06. l. Cell cycle assays showed the cell cycle distributions of SYNS05 and SYNS06 after circRNF10 overexpression. Data are shown as the mean ± SD (three independent experiments). *p < 0.05; **p < 0.01; ***p < 0.001; ns, no significance.

**Additional file 3: Fig. S3. **CircRNF10 silencing inhibits GSCs viability, proliferation, neurospheres formation and stemness. a. qPCR assays showed the expression of circRNF10 in SYNS03 and SYNS08 with circRNF10 knockdown. b, c. MTS assays showed the cell viabilities of circRNF10-silenced SYNS03 (b) and SYNS08 (c). d, e. Representative images of EdU assays showed the proliferation of circRNF10-silenced SYNS03 and SYNS08. Scale bar = 50μm. f-i, Representative images of NSFA with circRNF10 knockdown in SYNS03 and SYNS08 (f). Relative sizes of neurospheres of circRNF10-silenced SYNS03 and SYNS08 (g). Scale bar = 100μm. ELDA in SYNS03 (h) and SYNS08 (i) after circRNF10 downregulation. j. Western blotting showed the stemness markers expression in circRNF10-silenced SYNS03 and SYNS08. k. Cell cycle assays showed the cell cycle distributions of SYNS03 and SYNS08 after circRNF10 knockdown. Data are shown as the mean ± SD (three independent experiments). *p < 0.05; **p < 0.01; ***p < 0.001; ns, no significance.

**Additional file 4: Fig. S4. **Correlation analysis of circRNF10 and related genes based on clinical glioma tissue specimens. The correlation between circRNF10 and GPX4 (a), SLC7A11 (b), ZBTB48 (c), HSPB1 (d), IGF2BP3 (e) were determined from our glioma cohort. Data are shown as the mean ± SD (three independent experiments). *p < 0.05; **p < 0.01; ***p < 0.001; ns, no significance.

**Additional file 5: Fig. S5. **CircRNF10 promotes GSCs viability and proliferation in a ferroptosis-resistant manner in vitro. a, b. MTS assays showed the cell viabilities of circRNF10-silenced SYNS03 (a) and SYNS08 (b) followed by Fer-1 treatment. c, d. Representative images of EdU assays showed the proliferation of circRNF10-knockdown SYNS03 and SYNS08, followed by Fer-1 treatment. Scale bar = 100μm. e. Correlation analysis between GSVA-FerroScore and mRNA-si based on TCGA-GBMLGG dataset. f. Cell cycle assays showed the cell cycle distributions of circRNF10-knockdown SYNS03 and SYNS08 after Fer-1 treatment. Data are shown as the mean ± SD (three independent experiments). *p < 0.05; **p < 0.01; ***p < 0.001; ns, no significance.

**Additional file 6: Fig. S6. **CircRNF10 binds to and regulates the ZBTB48 protein expression. a. CircRNF10 binds to ZBTB48 proteins via CatRAPID prediction. b. RNA pull-down assays in SYNS05 displayed the circRNF10 probe pulled down ZBTB48 proteins. c-e. RIP assays showed anti-ZBTB48 treatment leaded to circRNF10 enrichment in circRNF10-silenced SYNS08 (c), circRNF10-overexpressed SYNS05 (d) and SYNS06 (e). f, g. Separate RIP assays for the nucleus and cytoplasm of SYNS03 (f) and SYNS05 (g) showed anti-ZBTB48 treatment leaded to circRNF10 enrichment in cytoplasm. h. qPCR assays of the mRNA expression of ZBTB48 in circRNF10-knockdown SYNS03 and SYNS08. i. Western blotting showed the protein level change of ZBTB48 after circRNF10 downregulation in SYNS03 and SYNS08. Data are presented as the mean ± SD (three independent experiments). *p < 0.05; **p < 0.01; ***p < 0.001; ns, no significance.

**Additional file 7: Fig. S7. **CircRNF10 binds to MKRN3 protein and prevents ZBTB48 degradation. a. RNA pulldown assays revealed the enriched MKRN3 pulled down by circRNF10 probe in SYNS05. b, c. Co-IP assays showed the interaction of ZBTB48 and MKRN3 in SYNS05. d, e. RIP assays displayed anti-MKRN3 treatment caused circRNF10 enrichment in circRNF10-overexpressed SYNS06 (d) and circRNF10-silenced SYNS08 (e). f. In vivo ubiquitination assays of polyubiquitin chains of ZBTB48 in SYNS03 transfected with wild-type, K27R, K29R or K33R mutant ubiquitin plasmids. Data are shown as the mean ± SD (three independent experiments). *p < 0.05; **p < 0.01; ***p < 0.001; ns, no significance.

**Additional file 8: Fig. S8. **ZBTB48 is upregulated and confers ferroptosis resistance in glioma. a-h. The expression of PRRX2 in different WHO grades (a, b), IDH status (c, d), 1p19q status (e, f) and MGMT status (g, h) in TCGA-glioma and CGGA datasets. i, j. The prognostic significance of ZBTB48 was verified in the TCGA (i), CGGA (j) databases. k, l. MDA(a) and GSH(b) contents detected in SYNS05 and SYNS06 with ZBTB48 overexpression. m, n. Lipid peroxidation levels (m) detected by BODIPY (581/591) C11 probe in ZBTB48-overexpressed SYNS05 and SYNS06. The relative fluorescence intensity of R-BODIPY quantified by Image J (n). Scale bar=100μm. Data are shown as the mean ± SD (three independent experiments). *p < 0.05; **p < 0.01; ***p < 0.001; ns, no significance.

**Additional file 9: Fig. S9. **ZBTB48 inhibits ferroptosis mediated by circRNF10-silenced in GSCs. a, b. MDA(a) and GSH(b) contents measured in SYNS03 and SYNS08 with circRNF10 knockdown followed by ZBTB48 overexpression. c, d. Lipid peroxidation levels (c) detected by BODIPY (581/591) C11 probe in circRNF10-knockdown SYNS03 and SYNS08 followed by ZBTB48 overexpression and the relative fluorescence intensity of O-BODIPY quantified by Image J (d). Scale bar=100μm. e, f. MTS assays showed the cell viabilities of circRNF10-silenced SYNS03 (e) and SYNS08 (f) followed by ZBTB48 overexpression. g-j, Representative images (g) of NSFA with circRNF10 knockdown in SYNS03 and SYNS08 followed by ZBTB48 upregulation. Scale bar = 200μm. ELDA in SYNS03 (h) and SYNS08 (i) after circRNF10 silencing followed by ZBTB48 overexpression. Relative sizes of neurospheres of circRNF10-silenced SYNS03 and SYNS08 after ZBTB48 overexpression (j). k, l. Representative images of EdU assays showed the proliferation of circRNF10-knockdown SYNS03 and SYNS08, followed by ZBTB48 overexpression. Scale bar = 100μm. m. Cell cycle assays showed the cell cycle distributions of SYNS03 and SYNS08 after circRNF10 knockdown with ZBTB48 overexpression. Data are shown as the mean ± SD (three independent experiments). *p < 0.05; **p < 0.01; ***p < 0.001; ns, no significance.

**Additional file 10: Fig. S10. **CircRNF10 promotes viability and proliferation of GSCs via upregulating HSPB1. a, b. MTS assays showed the cell viabilities of circRNF10-silenced SYNS03 (a) and SYNS08 (b) followed by HSPB1 overexpression. c, d. Representative images of EdU assays showed the proliferation of circRNF10-knockdown SYNS03 and SYNS08, followed by HSPB1 overexpression. Scale bar = 100μm. Data are shown as the mean ± SD (three independent experiments). *p < 0.05; **p < 0.01; ***p < 0.001; ns, no significance.

**Additional file 11: Fig. S11. **ZBTB48 transcriptionally upregulates IGF2BP3 to maintain stability of circRNF10. a, b. RIP assays showing anti-IGF2BP3 treatment enriched with circRNF10 after circRNF10 knockdown in SYNS08 (a) or overexpression in SYNS06 (b). c, d. RNA stability assays showing the half-life of circDNF10 in IGF2BP3- overexpressed (c) or silencing (d) GSCs followed by actinomycin D treatment. e, f. The Dual-luciferase reporter assays revealed the luciferase promoter activities of IGF2BP3 with ZBTB48 silencing in SYNS08 (e) and ZBTB48 overexpression in SYNS06 (f). g. The ChIP qPCR showing the enrichment difference of IGF2BP3 promoter sequence via anti-ZBTB48 treatment in ZBTB48- overexpressed SYNS05 and SYNS06. Data are shown as the mean ± SD (three independent experiments). *p < 0.05; **p < 0.01; ***p < 0.001; ns, no significance.

**Additional file 12: Fig. S12. **The expression of ZBTB48, MKRN3, HSPB1 and IGF2BP3 in GSCs after lentiviral-based transfection. a-d. The western blot and qPCR assays investigated the alterations in both transcriptional and translational levels of ZBTB48 (a), MKRN3(b), HSPB1(c) and IGF2BP3(d) following intervention through molecular biology techniques in GSCs. Data are shown as the mean ± SD (three independent experiments). *p < 0.05; **p < 0.01; ***p < 0.001; ns, no significance.

**Additional file 13.**

## Data Availability

The analyzed data sets generated during the present study are available from the corresponding author on reasonable request.

## Abbreviations

NBT: Normal brain tissues
GSCs: Glioma stem cells
GBM: Glioblastoma
circRNAs: Circular RNAs
GSH: Glutathione
MDA: Malondialdehyde
TF: Transcription factor
RBPs: RNA binding proteins
FISH: Fluorescence in situ hybridization
qRT-PCR: Real-time quantitative reverse transcription PCR
RIP: RNA immunoprecipitation
ChIP: Chromatin immunoprecipitation
IHC: Immunohistochemistry
MTS: 3-(4, 5-Dimethylthiazol-2-yl)-5-(3-carboxymethoxyphenyl)-2-(4-sulfophenyl)-2H tetrazolium
EdU: 5-Ethynyl-20-deoxyuridine
NFSA: Neurosphere formation assay
ELDA: Extreme Limiting Dilution Analysis
RCD: Regulated cell death
ROS: Reactive oxygen species
WHO: World Health Organization
STR: Short tandem repeat
O-BODIPY: Oxidized BODIPY
R-BODIPY: Reduced BODIPY
TBA: Thiobarbituric acid
GIS: German immunohistochemical score
GSEA: Gene set enrichment analysis
TCGA: The Cancer Genome Atlas
CGGA: Chinese Glioma Genome Atlas
GEO: Gene Expression Omnibus
PCD: Programmed cell death
