# Supplementary figures and images for: CircRNF10 triggers a positive feedback loop to facilitate progression of glioblastoma via redeploying the ferroptosis defense in GSCs
Source: J Exp Clin Cancer Res. 2023 Sep 19;42:242. doi: 10.1186/s13046-023-02816-9 (PMC10507871; doi:10.1186/s13046-023-02816-9)

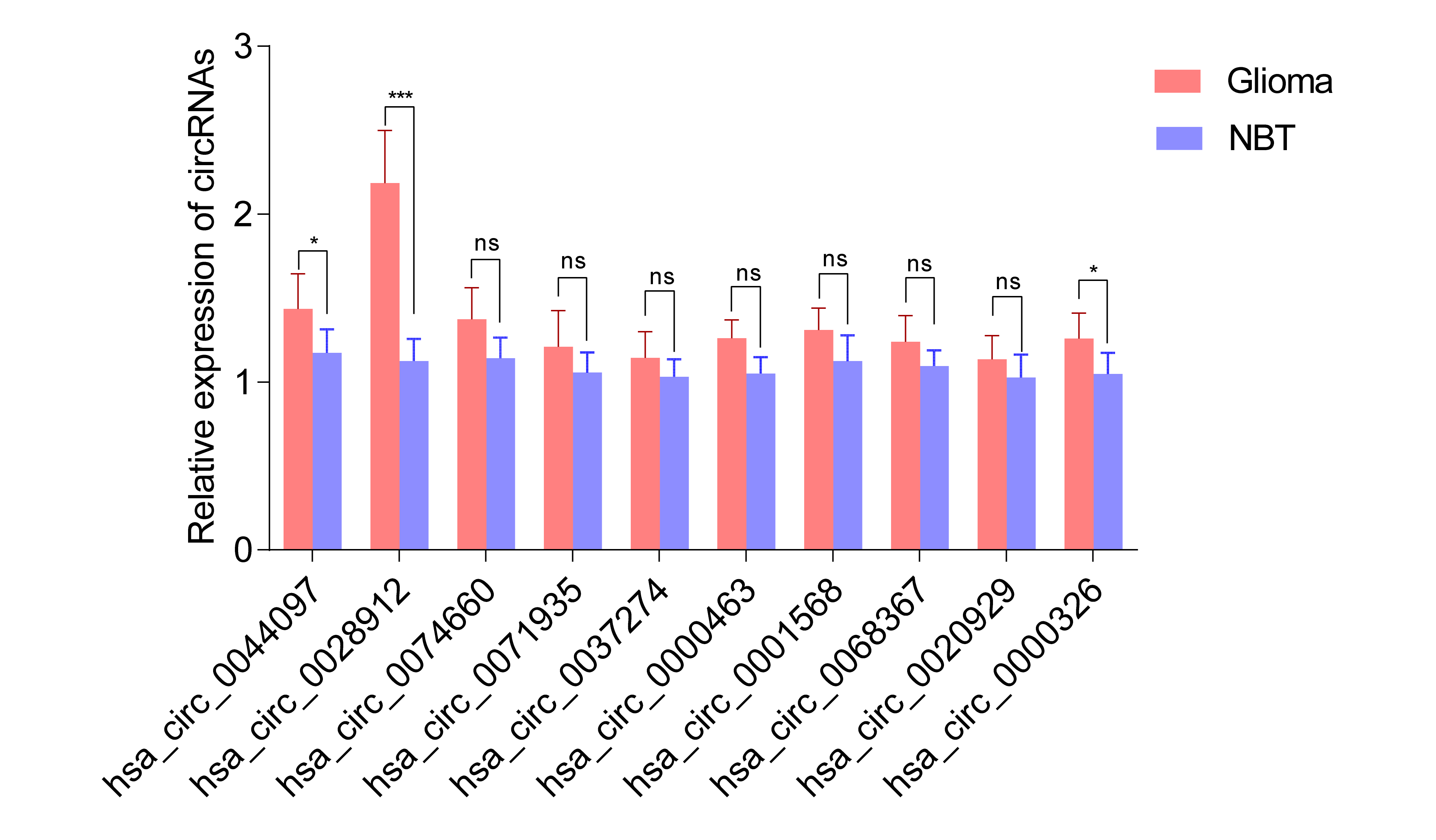

Supplement: Supplementary file 1 — Additional file 1: Fig. S1. Validation of the expression of the top 10 upregulated circRNAs in glioma and normal brain tissues (NBT). qPCR displays the expression of top 10 circRNAs in glioma and NBT. All data are expressed as the mean ± SD (three independent experiments). *p < 0.05; **p < 0.01; ***p < 0.001; ns, no significance. [file 13046_2023_2816_MOESM1_ESM.tif]

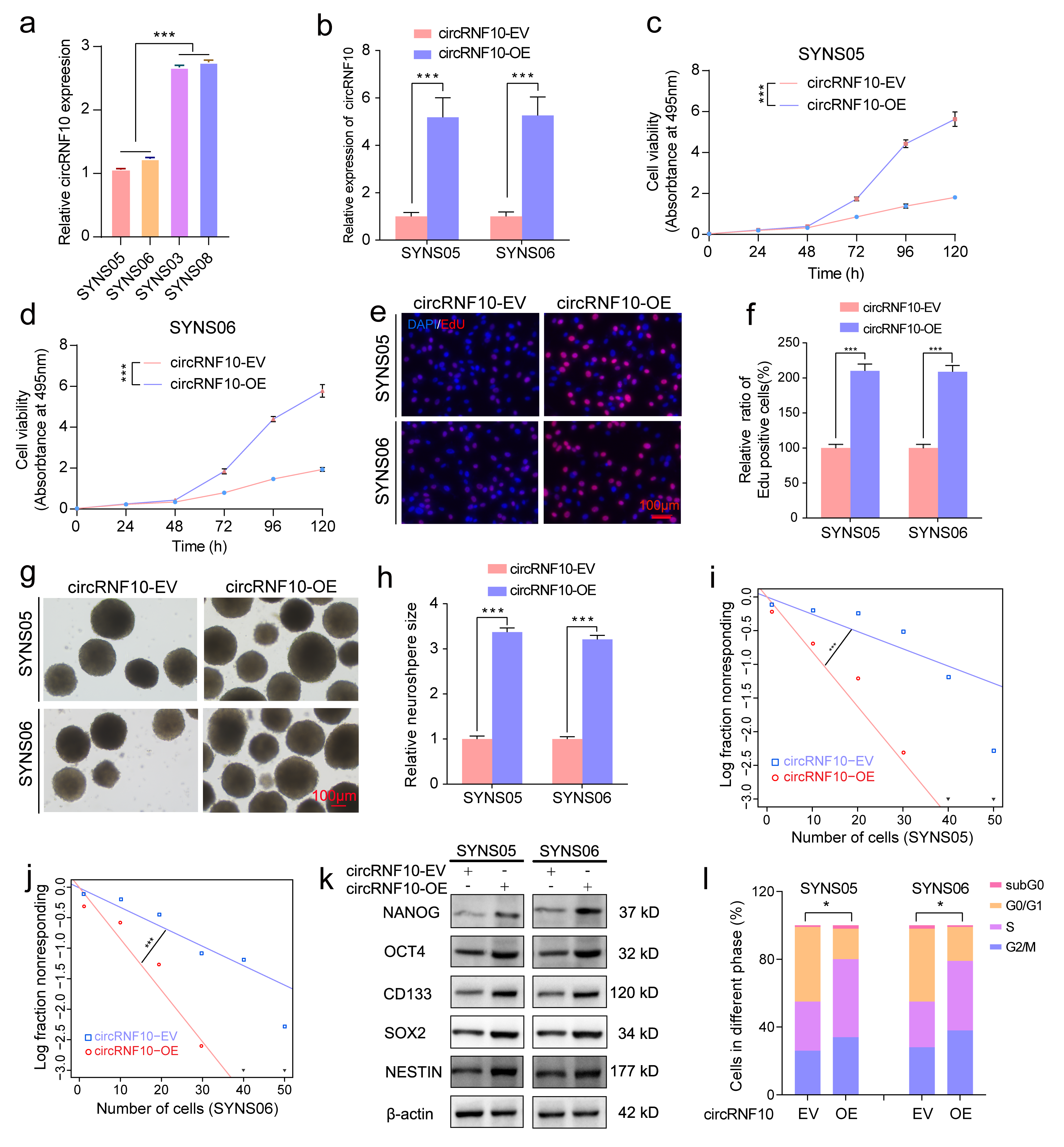

Supplement: Supplementary file 2 — Additional file 2: Fig. S2. CircRNF10 overexpression promotes viability, proliferation, neurospheres formation and stemness of GSCs. a. qPCR showed the expression of circRNF10 in GSCs derived from GBM patients. b. qPCR assays displayed the expression of circRNF10 in SYNS05 and SYNS06 after circRNF10 overexpression. c, d. MTS assays showed the cell viabilities of circRNF10-upregulated SYNS05 (c) and SYNS06 (d). e, f. Representative images of EdU assays showed the proliferation of circRNF10-overexpressed SYNS05 and SYNS06. Scale bar = 100μm. g-j. Representative images of NSFA with circRNF10 overexpression in SYNS05 and SYNS06 (g). Relative sizes of neurospheres of circRNF10-upregulated SYNS05 and SYNS06 (h). Scale bar = 100μm. ELDA in SYNS05 (i) and SYNS06 (j) after circRNF10 overexpression. k. Western blotting showed the stemness markers expression in circRNF10-overexpressed SYNS05 and SYNS06. l. Cell cycle assays showed the cell cycle distributions of SYNS05 and SYNS06 after circRNF10 overexpression. Data are shown as the mean ± SD (three independent experiments). *p < 0.05; **p < 0.01; ***p < 0.001; ns, no significance. [file 13046_2023_2816_MOESM2_ESM.tif]

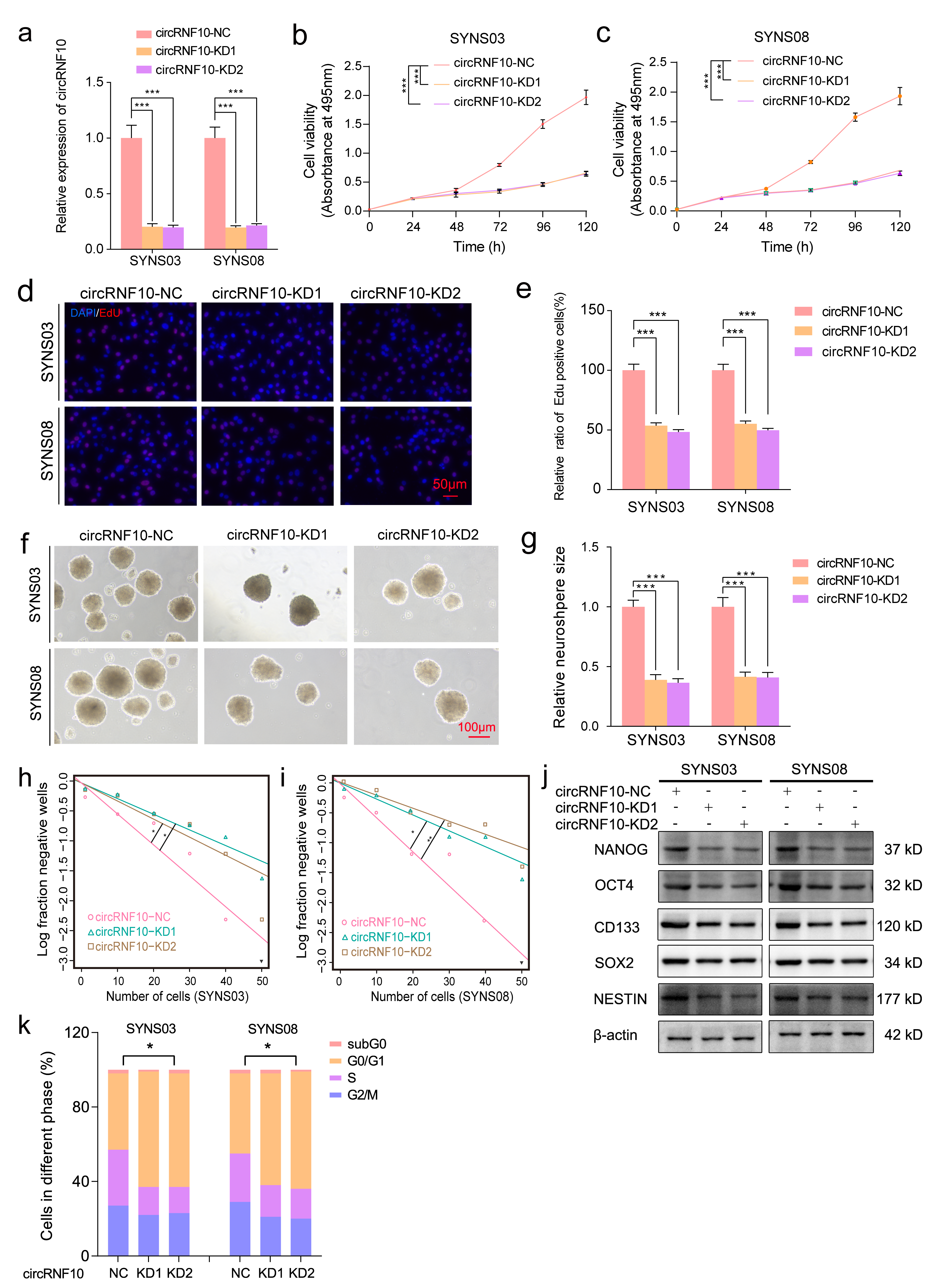

Supplement: Supplementary file 3 — Additional file 3: Fig. S3. CircRNF10 silencing inhibits GSCs viability, proliferation, neurospheres formation and stemness. a. qPCR assays showed the expression of circRNF10 in SYNS03 and SYNS08 with circRNF10 knockdown. b, c. MTS assays showed the cell viabilities of circRNF10-silenced SYNS03 (b) and SYNS08 (c). d, e. Representative images of EdU assays showed the proliferation of circRNF10-silenced SYNS03 and SYNS08. Scale bar = 50μm. f-i, Representative images of NSFA with circRNF10 knockdown in SYNS03 and SYNS08 (f). Relative sizes of neurospheres of circRNF10-silenced SYNS03 and SYNS08 (g). Scale bar = 100μm. ELDA in SYNS03 (h) and SYNS08 (i) after circRNF10 downregulation. j. Western blotting showed the stemness markers expression in circRNF10-silenced SYNS03 and SYNS08. k. Cell cycle assays showed the cell cycle distributions of SYNS03 and SYNS08 after circRNF10 knockdown. Data are shown as the mean ± SD (three independent experiments). *p < 0.05; **p < 0.01; ***p < 0.001; ns, no significance. [file 13046_2023_2816_MOESM3_ESM.tif]

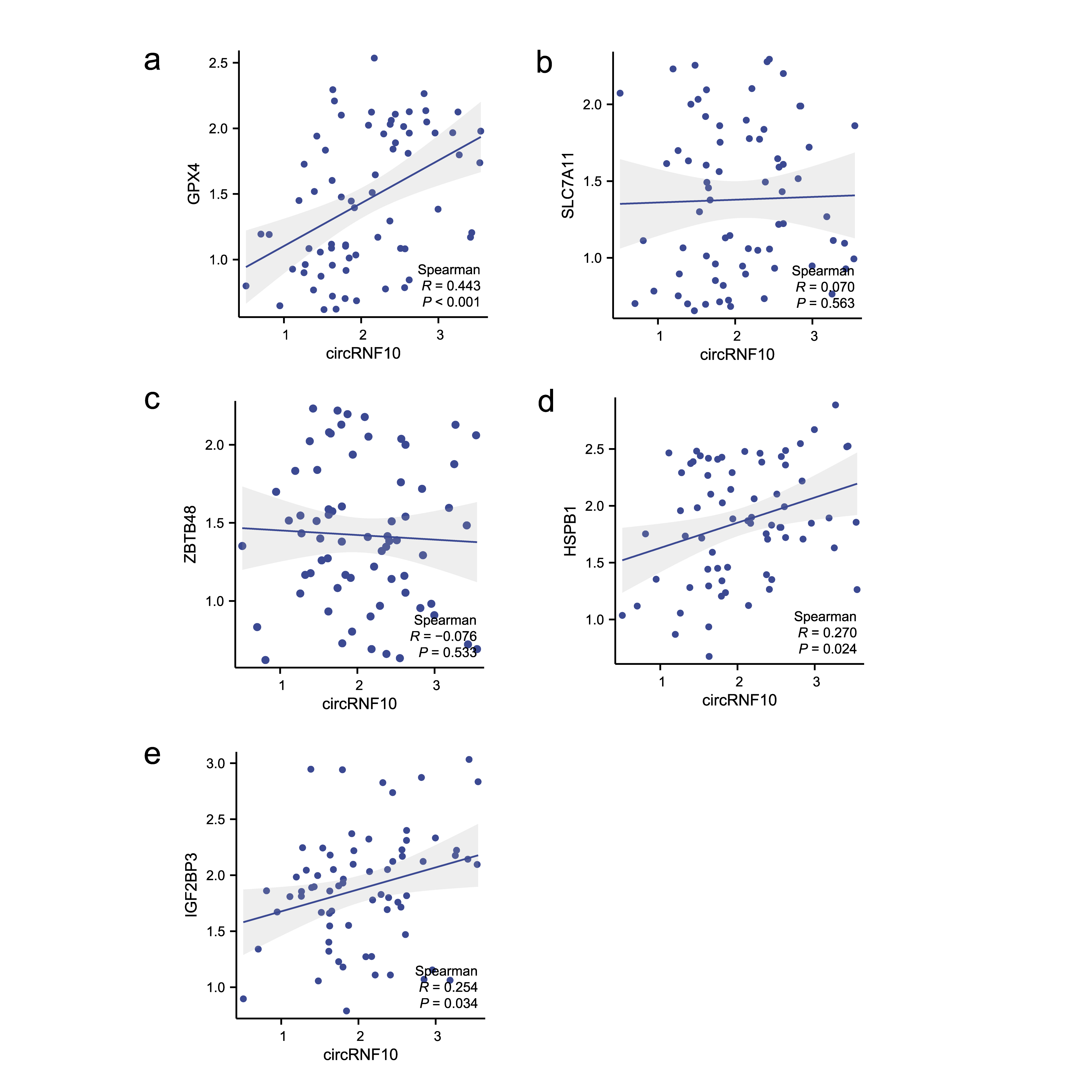

Supplement: Supplementary file 4 — Additional file 4: Fig. S4. Correlation analysis of circRNF10 and related genes based on clinical glioma tissue specimens. The correlation between circRNF10 and GPX4 (a), SLC7A11 (b), ZBTB48 (c), HSPB1 (d), IGF2BP3 (e) were determined from our glioma cohort. Data are shown as the mean ± SD (three independent experiments). *p < 0.05; **p < 0.01; ***p < 0.001; ns, no significance. [file 13046_2023_2816_MOESM4_ESM.tif]

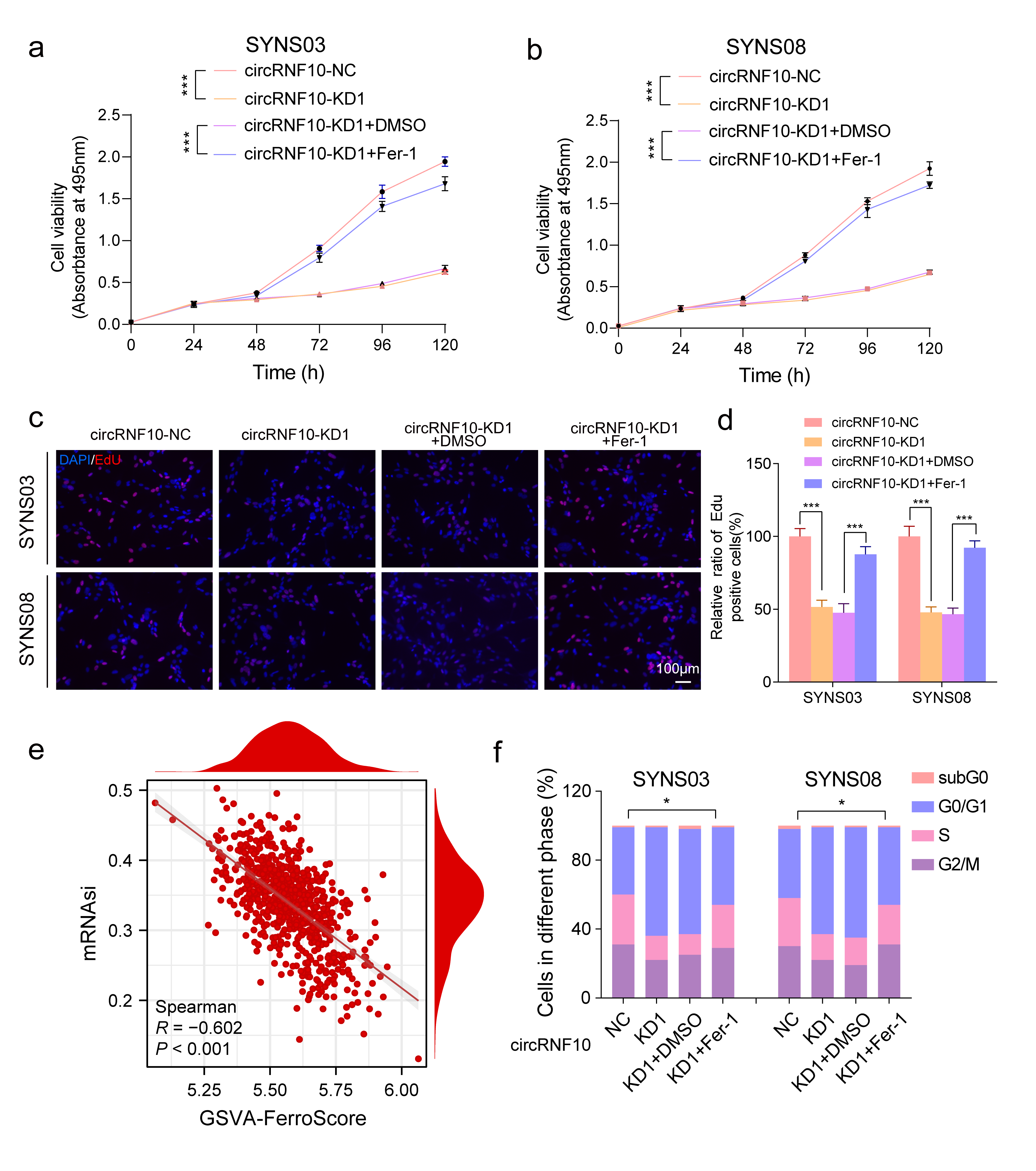

Supplement: Supplementary file 5 — Additional file 5: Fig. S5. CircRNF10 promotes GSCs viability and proliferation in a ferroptosis-resistant manner in vitro. a, b. MTS assays showed the cell viabilities of circRNF10-silenced SYNS03 (a) and SYNS08 (b) followed by Fer-1 treatment. c, d. Representative images of EdU assays showed the proliferation of circRNF10-knockdown SYNS03 and SYNS08, followed by Fer-1 treatment. Scale bar = 100μm. e. Correlation analysis between GSVA-FerroScore and mRNA-si based on TCGA-GBMLGG dataset. f. Cell cycle assays showed the cell cycle distributions of circRNF10-knockdown SYNS03 and SYNS08 after Fer-1 treatment. Data are shown as the mean ± SD (three independent experiments). *p < 0.05; **p < 0.01; ***p < 0.001; ns, no significance. [file 13046_2023_2816_MOESM5_ESM.tif]

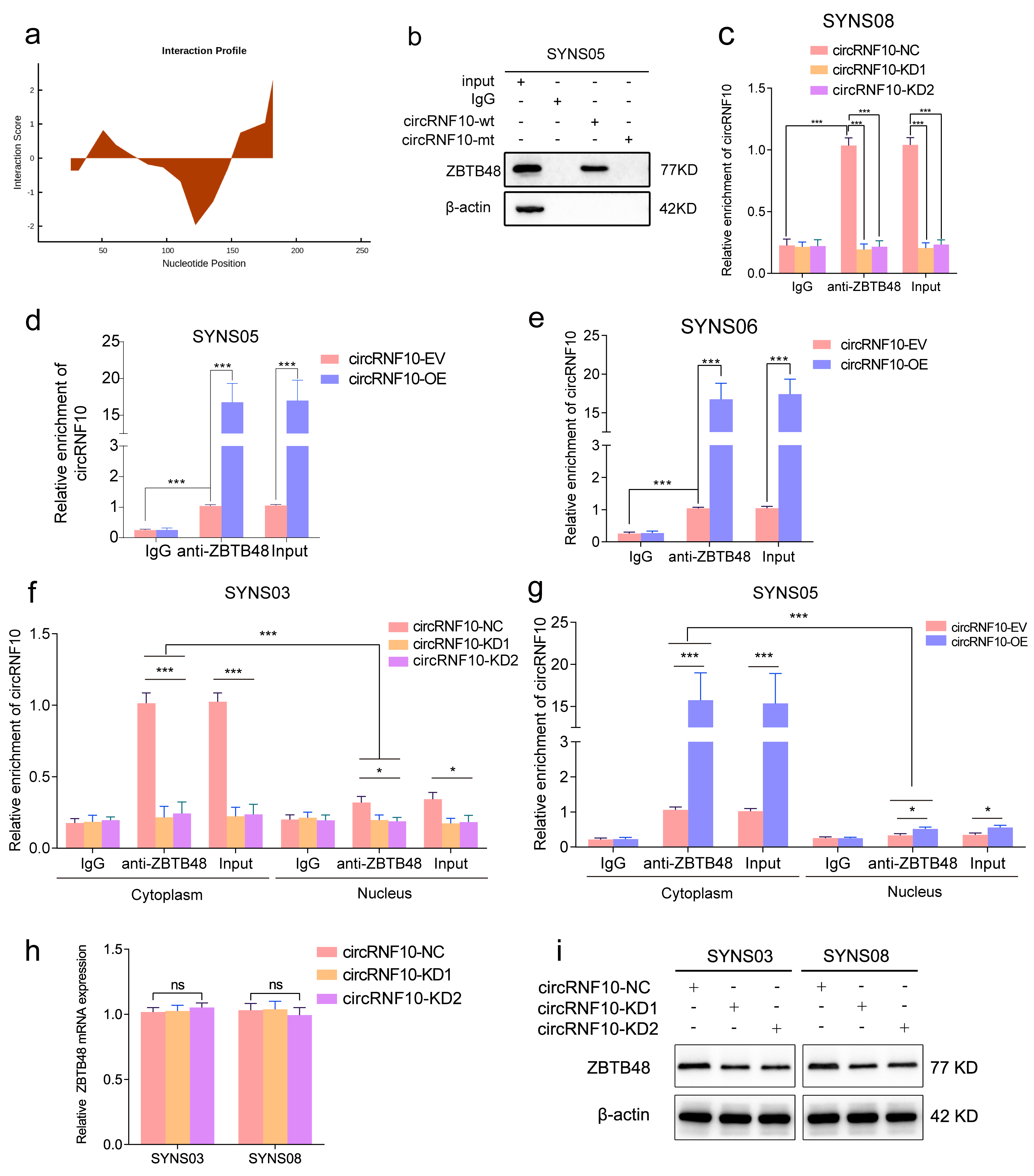

Supplement: Supplementary file 6 — Additional file 6: Fig. S6. CircRNF10 binds to and regulates the ZBTB48 protein expression. a. CircRNF10 binds to ZBTB48 proteins via CatRAPID prediction. b. RNA pull-down assays in SYNS05 displayed the circRNF10 probe pulled down ZBTB48 proteins. c-e. RIP assays showed anti-ZBTB48 treatment leaded to circRNF10 enrichment in circRNF10-silenced SYNS08 (c), circRNF10-overexpressed SYNS05 (d) and SYNS06 (e). f, g. Separate RIP assays for the nucleus and cytoplasm of SYNS03 (f) and SYNS05 (g) showed anti-ZBTB48 treatment leaded to circRNF10 enrichment in cytoplasm. h. qPCR assays of the mRNA expression of ZBTB48 in circRNF10-knockdown SYNS03 and SYNS08. i. Western blotting showed the protein level change of ZBTB48 after circRNF10 downregulation in SYNS03 and SYNS08. Data are presented as the mean ± SD (three independent experiments). *p < 0.05; **p < 0.01; ***p < 0.001; ns, no significance. [file 13046_2023_2816_MOESM6_ESM.tif]

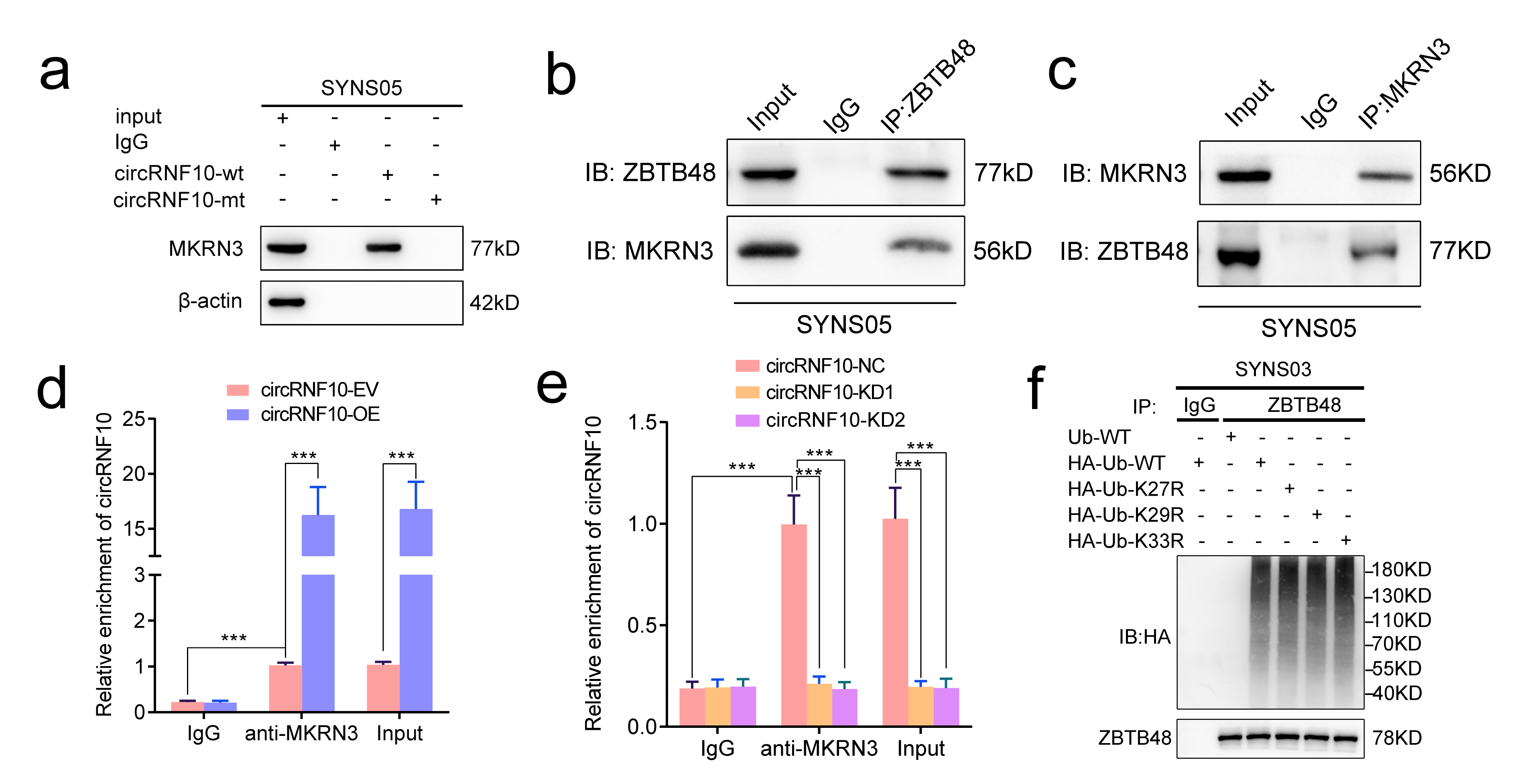

Supplement: Supplementary file 7 — Additional file 7: Fig. S7. CircRNF10 binds to MKRN3 protein and prevents ZBTB48 degradation. a. RNA pulldown assays revealed the enriched MKRN3 pulled down by circRNF10 probe in SYNS05. b, c. Co-IP assays showed the interaction of ZBTB48 and MKRN3 in SYNS05. d, e. RIP assays displayed anti-MKRN3 treatment caused circRNF10 enrichment in circRNF10-overexpressed SYNS06 (d) and circRNF10-silenced SYNS08 (e). f. In vivo ubiquitination assays of polyubiquitin chains of ZBTB48 in SYNS03 transfected with wild-type, K27R, K29R or K33R mutant ubiquitin plasmids. Data are shown as the mean ± SD (three independent experiments). *p < 0.05; **p < 0.01; ***p < 0.001; ns, no significance. [file 13046_2023_2816_MOESM7_ESM.tif]

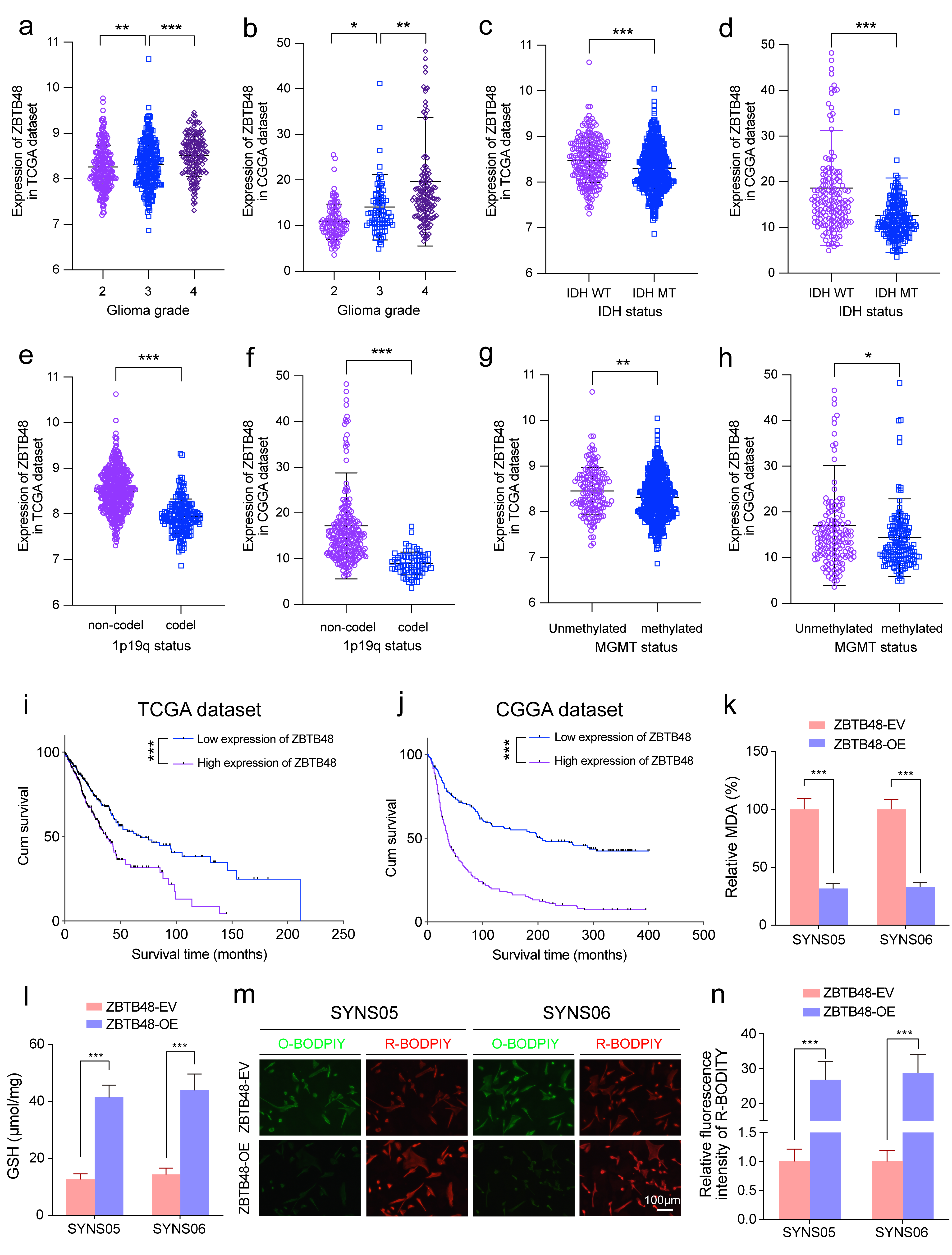

Supplement: Supplementary file 8 — Additional file 8: Fig. S8. ZBTB48 is upregulated and confers ferroptosis resistance in glioma. a-h. The expression of PRRX2 in different WHO grades (a, b), IDH status (c, d), 1p19q status (e, f) and MGMT status (g, h) in TCGA-glioma and CGGA datasets. i, j. The prognostic significance of ZBTB48 was verified in the TCGA (i), CGGA (j) databases. k, l. MDA(a) and GSH(b) contents detected in SYNS05 and SYNS06 with ZBTB48 overexpression. m, n. Lipid peroxidation levels (m) detected by BODIPY (581/591) C11 probe in ZBTB48-overexpressed SYNS05 and SYNS06. The relative fluorescence intensity of R-BODIPY quantified by Image J (n). Scale bar=100μm. Data are shown as the mean ± SD (three independent experiments). *p < 0.05; **p < 0.01; ***p < 0.001; ns, no significance. [file 13046_2023_2816_MOESM8_ESM.tif]

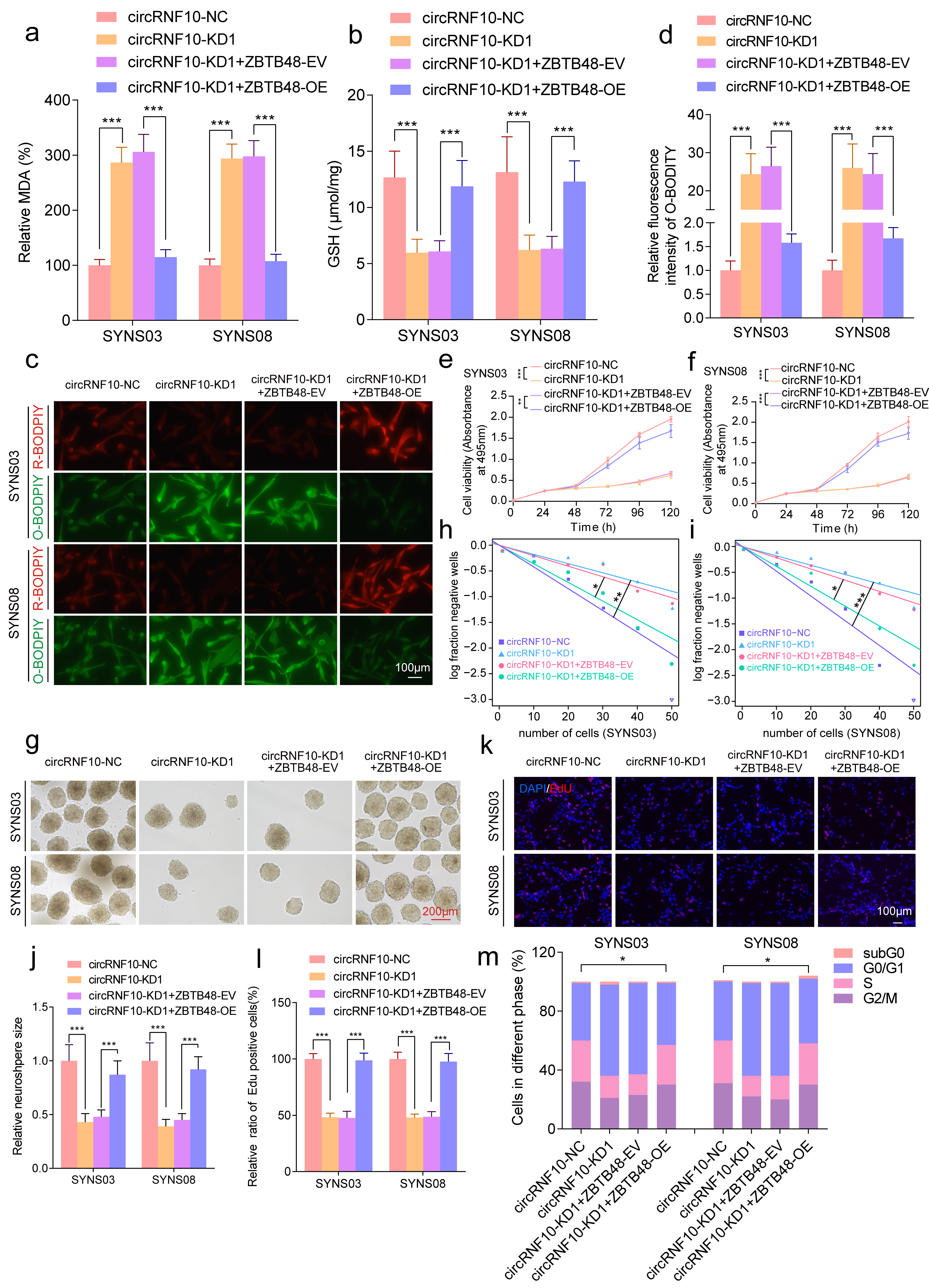

Supplement: Supplementary file 9 — Additional file 9: Fig. S9. ZBTB48 inhibits ferroptosis mediated by circRNF10-silenced in GSCs. a, b. MDA(a) and GSH(b) contents measured in SYNS03 and SYNS08 with circRNF10 knockdown followed by ZBTB48 overexpression. c, d. Lipid peroxidation levels (c) detected by BODIPY (581/591) C11 probe in circRNF10-knockdown SYNS03 and SYNS08 followed by ZBTB48 overexpression and the relative fluorescence intensity of O-BODIPY quantified by Image J (d). Scale bar=100μm. e, f. MTS assays showed the cell viabilities of circRNF10-silenced SYNS03 (e) and SYNS08 (f) followed by ZBTB48 overexpression. g-j, Representative images (g) of NSFA with circRNF10 knockdown in SYNS03 and SYNS08 followed by ZBTB48 upregulation. Scale bar = 200μm. ELDA in SYNS03 (h) and SYNS08 (i) after circRNF10 silencing followed by ZBTB48 overexpression. Relative sizes of neurospheres of circRNF10-silenced SYNS03 and SYNS08 after ZBTB48 overexpression (j). k, l. Representative images of EdU assays showed the proliferation of circRNF10-knockdown SYNS03 and SYNS08, followed by ZBTB48 overexpression. Scale bar = 100μm. m. Cell cycle assays showed the cell cycle distributions of SYNS03 and SYNS08 after circRNF10 knockdown with ZBTB48 overexpression. Data are shown as the mean ± SD (three independent experiments). *p < 0.05; **p < 0.01; ***p < 0.001; ns, no significance. [file 13046_2023_2816_MOESM9_ESM.tif]

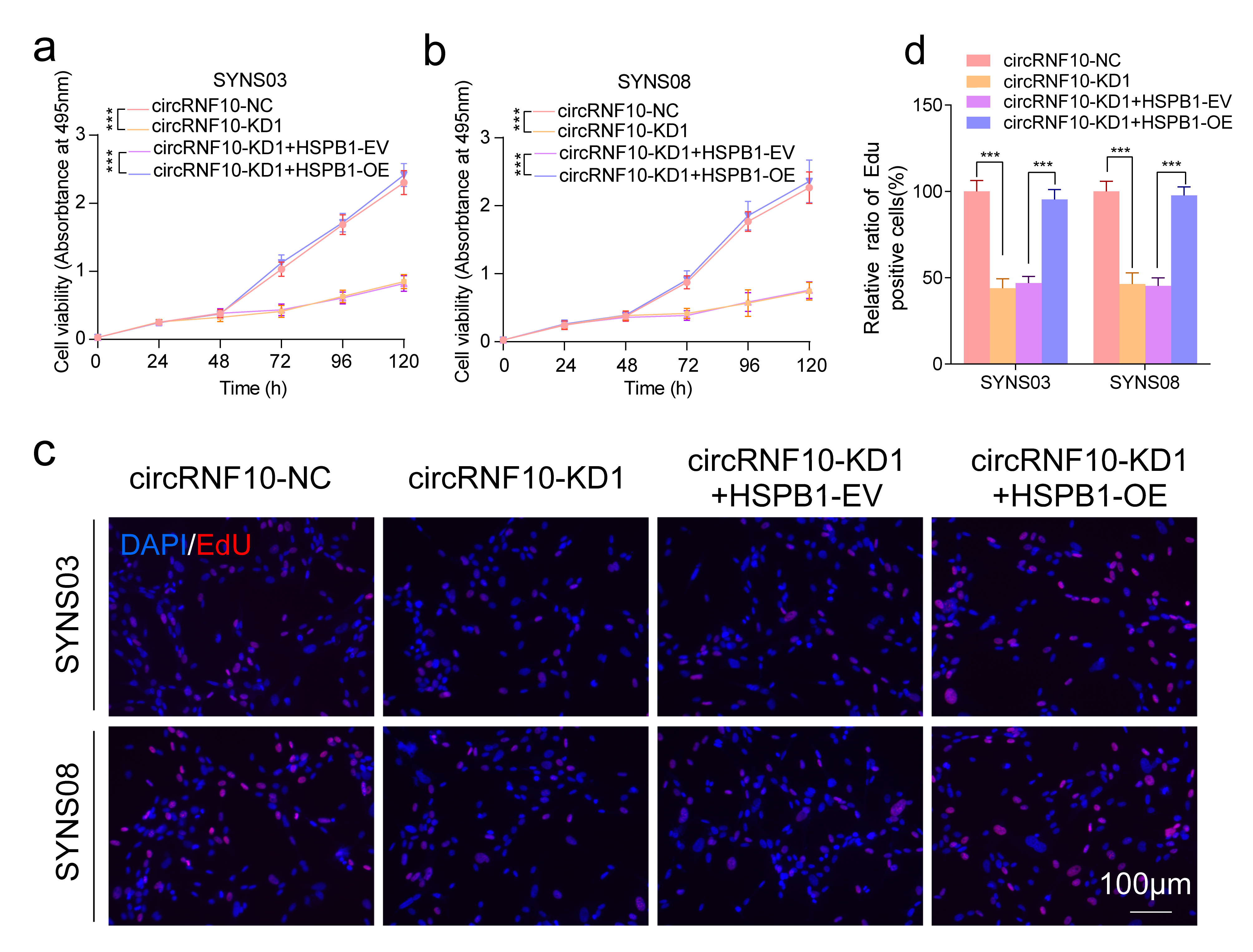

Supplement: Supplementary file 10 — Additional file 10: Fig. S10. CircRNF10 promotes viability and proliferation of GSCs via upregulating HSPB1. a, b. MTS assays showed the cell viabilities of circRNF10-silenced SYNS03 (a) and SYNS08 (b) followed by HSPB1 overexpression. c, d. Representative images of EdU assays showed the proliferation of circRNF10-knockdown SYNS03 and SYNS08, followed by HSPB1 overexpression. Scale bar = 100μm. Data are shown as the mean ± SD (three independent experiments). *p < 0.05; **p < 0.01; ***p < 0.001; ns, no significance. [file 13046_2023_2816_MOESM10_ESM.tif]

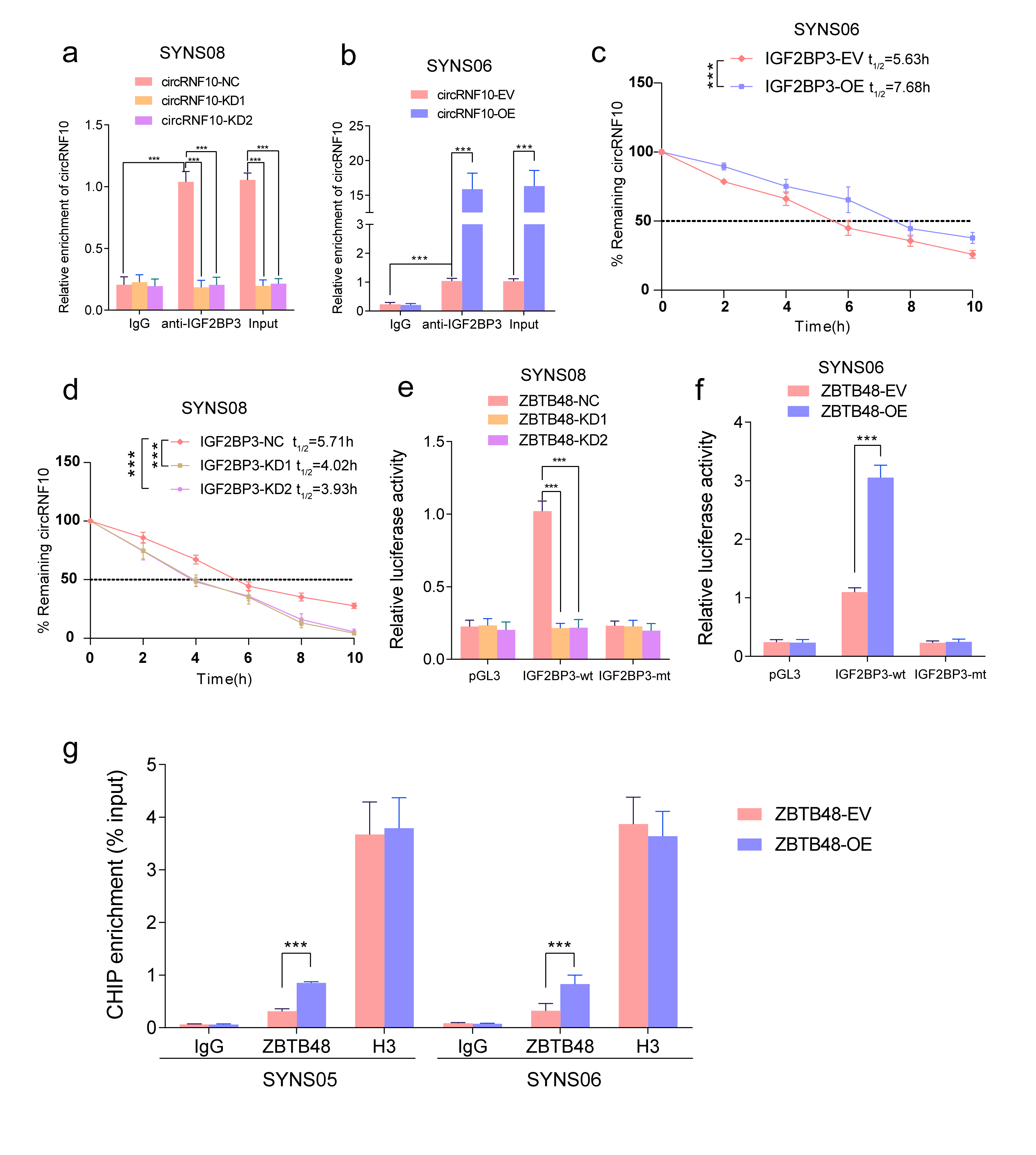

Supplement: Supplementary file 11 — Additional file 11: Fig. S11. ZBTB48 transcriptionally upregulates IGF2BP3 to maintain stability of circRNF10. a, b. RIP assays showing anti-IGF2BP3 treatment enriched with circRNF10 after circRNF10 knockdown in SYNS08 (a) or overexpression in SYNS06 (b). c, d. RNA stability assays showing the half-life of circDNF10 in IGF2BP3- overexpressed (c) or silencing (d) GSCs followed by actinomycin D treatment. e, f. The Dual-luciferase reporter assays revealed the luciferase promoter activities of IGF2BP3 with ZBTB48 silencing in SYNS08 (e) and ZBTB48 overexpression in SYNS06 (f). g. The ChIP qPCR showing the enrichment difference of IGF2BP3 promoter sequence via anti-ZBTB48 treatment in ZBTB48- overexpressed SYNS05 and SYNS06. Data are shown as the mean ± SD (three independent experiments). *p < 0.05; **p < 0.01; ***p < 0.001; ns, no significance. [file 13046_2023_2816_MOESM11_ESM.tif]

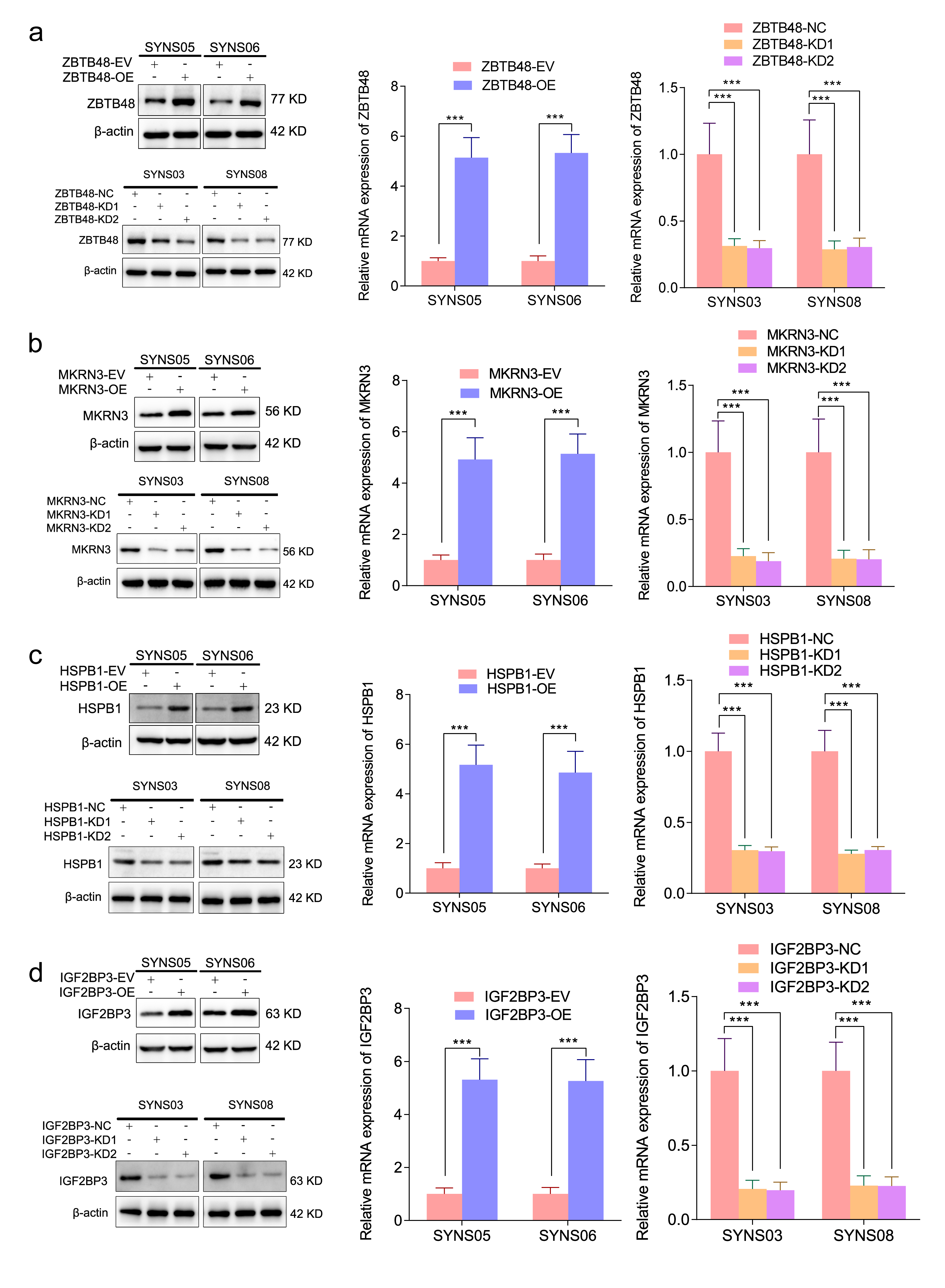

Supplement: Supplementary file 12 — Additional file 12: Fig. S12. The expression of ZBTB48, MKRN3, HSPB1 and IGF2BP3 in GSCs after lentiviral-based transfection. a-d. The western blot and qPCR assays investigated the alterations in both transcriptional and translational levels of ZBTB48 (a), MKRN3(b), HSPB1(c) and IGF2BP3(d) following intervention through molecular biology techniques in GSCs. Data are shown as the mean ± SD (three independent experiments). *p < 0.05; **p < 0.01; ***p < 0.001; ns, no significance. [file 13046_2023_2816_MOESM12_ESM.tif]
